# Fortified Eggs as Food-Based Vehicles for Nutrient Delivery: A Scoping Review of Human Intervention Studies

**DOI:** 10.3390/nu18132189

**Published:** 2026-07-05

**Authors:** Liusen Wang, Hongru Jiang, Weiyi Li, Lixin Hao, Ziyan Liu, Xu Yan, Jingming Yang, Yang Liu, Chao Gao

**Affiliations:** 1 National Institute for Nutrition and Health, Chinese Center for Disease Control and Prevention, Beijing 100050, China; wangls@ninh.chinacdc.cn (L.W.); jianghr@ninh.chinacdc.cn (H.J.); liwy@ninh.chinacdc.cn (W.L.); haolx@ninh.chinacdc.cn (L.H.); yangjm@ninh.chinacdc.cn (J.Y.); liuyang@ninh.chinacdc.cn (Y.L.); 2 Key Laboratory of Public Nutrition and Health, National Health Commission of the People’s Republic of China, Beijing 100050, China; 3 College of Biochemical Engineering, Beijing Union University, Beijing 100101, China; 16619799423@163.com (Z.L.); 13269029012@163.com (X.Y.)

**Keywords:** fortified eggs, functional foods, nutrient delivery, micronutrient deficiency, *n*-3 fatty acids, carotenoids, iodine, evidence mapping

## Abstract

**Background/Objectives**: Fortified eggs have been proposed as food-based vehicles for delivering micronutrients and other bioactive compounds. However, human intervention evidence remains fragmented across nutrient targets, populations, intervention designs, and outcome domains. This review mapped human intervention studies and summarized the reported nutritional and health-related outcomes associated with fortified egg consumption. **Methods**: PubMed, Embase, Web of Science, the Cochrane Library, CNKI, Wanfang Data, and SinoMed were searched for studies published in English or Chinese from 1 January 2005 to 1 June 2025. Eligible studies were human intervention studies evaluating fortified, enriched, or bio-enhanced eggs and reporting at least one nutritional or health-related outcome. Owing to substantial heterogeneity in nutrient type, intervention design, comparator, duration, and outcome assessment, findings were synthesized narratively. **Results**: Thirty-seven human intervention studies met the eligibility criteria. Most evaluated eggs enriched with *n*-3 polyunsaturated fatty acids, followed by carotenoid-enriched eggs, whereas relatively few examined iodine or other fortified nutrients. Across studies, fortified egg consumption was generally associated with improvements in intake estimates and biomarkers of targeted nutrient status, including circulating fatty acids, serum carotenoids, and, in a limited number of studies, urinary iodine. Evidence for downstream health-related outcomes was more limited, inconsistent, and difficult to compare across nutrient categories and study populations. **Conclusions**: Fortified eggs may serve as useful food-based vehicles for improving the delivery of selected nutrients and short-term nutrient-status biomarkers. However, the evidence base remains heterogeneous and nutrient-specific, and it is still uncertain whether these changes translate into consistent, clinically meaningful health benefits. Larger, longer-term, and more rigorously reported trials, particularly in populations at nutritional risk, are needed.

## 1. Introduction

Eggs are widely regarded as nutrient-dense foods that provide high-quality protein and a range of essential micronutrients, including vitamins A, D, E, and B12, as well as choline, selenium, and iodine [1]. Owing to their affordability, culinary versatility, and widespread consumption, eggs contribute importantly to dietary quality across diverse populations and dietary patterns [2]. Eggs have also long been discussed in relation to dietary cholesterol, overall diet quality, and the development of functional foods, making them a particularly visible and contested food in nutrition research and policy. This broader nutritional and public health context partly explains the sustained interest in modifying egg composition for targeted nutritional purposes. At the same time, the nutritional value of eggs in their conventional form does not necessarily address specific micronutrient gaps that remain common at the population level. Inadequate iodine intake, low consumption of long-chain *n*-3 polyunsaturated fatty acids (PUFAs), and suboptimal carotenoid status continue to be important public health concerns and are associated with adverse outcomes across the life course [3,4,5,6,7]. In this context, food fortification remains an established strategy for improving nutrient intake and nutritional status, particularly when implemented through foods that are already commonly consumed [8].

Among potential food vehicles, eggs are of particular interest because their nutrient composition can be intentionally modified during production, most commonly through dietary manipulation of laying hens, allowing targeted enrichment of selected nutrients without requiring major changes in consumer eating habits [9,10]. In practice, fortified eggs are typically produced through changes in hen feed composition, but the efficiency with which different nutrients are transferred and deposited into the edible egg may vary substantially according to the nutrient used, feed formulation, and production conditions. In addition, the post-deposition stability of fortified nutrients during production, storage, and preparation may not be uniform across fortification strategies [11,12]. Depending on the nutrient of interest, feed-based fortification can increase the deposition of compounds such as iodine, selenium, *n*-3 PUFAs, vitamin D, lutein, or zeaxanthin into the edible egg, although the efficiency, stability, and consistency of deposition may vary across nutrients and production systems [10]. This production pathway distinguishes fortified eggs from conventional eggs and supports their consideration as a food-based nutrient-delivery platform rather than simply as a naturally nutrient-rich food [13].

Fortified or enriched eggs have been developed to increase the concentrations of selected nutrients, most notably iodine [14], *n*-3 PUFAs [15], and carotenoids such as lutein and zeaxanthin [16,17,18]. These nutrients are of interest because of their relevance to thyroid function, cardiometabolic health, and visual health, respectively [14,19,20]. Importantly, however, these interventions should not be interpreted as a single, uniform nutritional strategy. Eggs enriched with iodine, *n*-3 PUFAs, carotenoids, vitamin D, or multiple nutrients differ in their biological rationale, target populations, intended outcomes, and degree of translational maturity [10,21,22]. What links them is not a shared mechanism of action, but a shared delivery platform: the egg as a familiar, widely accepted food in which nutrient composition can be modified during production and subsequently consumed in a relatively standardized form. Accordingly, the value of reviewing these studies together lies not in assuming equivalence across fortification strategies, but in examining how different nutrient-specific interventions have used the same food vehicle to address distinct nutritional questions.

In addition to serving as a carrier, the egg matrix itself may influence nutrient utilization. Its lipid and phospholipid components may facilitate the intestinal absorption of fat-soluble compounds, potentially enhancing the bioavailability of carotenoids and other lipophilic nutrients [23,24,25]. This feature has strengthened interest in fortified eggs as a practical food-based approach for delivering selected nutrients in a biologically relevant and consumer-familiar form. Nevertheless, the potential value of fortified eggs depends not only on nutrient enrichment itself, but also on whether the enriched nutrients are meaningfully absorbed, reflected in biomarkers of status, and ultimately associated with clinically relevant outcomes [26]. It also depends on whether nutrient enrichment is achieved in a technically robust and reproducible manner, an issue that has not always been clearly documented in the human intervention literature.

Over the past two decades, human intervention studies have examined fortified eggs across different populations and for a variety of purposes, from improving nutrient exposure or status to influencing cardiometabolic markers, thyroid-related indicators, or visual outcomes [14,17,20,23,27,28,29]. However, the evidence base remains fragmented. Studies differ substantially with respect to nutrient target, fortification approach, source or form of the nutrient used, reported egg nutrient level, intervention format, comparator, study duration, participant characteristics, and outcome assessment [13]. In some studies, the extent to which nutrient transfer or deposition efficiency was characterized is also unclear [30,31]. As a result, the literature is difficult to interpret as a coherent whole. At the same time, examining these studies together remains informative because it allows comparison of how different nutrient-specific egg interventions have been translated into human research, while also showing where evidence is concentrated, where it is sparse, and where cross-category generalization is not justified.

A broad evidence-mapping approach is therefore appropriate for this topic. Rather than attempting to determine a single pooled effect for “fortified eggs” as though they represent one homogeneous intervention, the present review aims to characterize the range and structure of the available human evidence, while preserving distinctions between nutrient categories and outcome domains [32]. Such an approach can help identify evidence clusters, clarify areas in which findings are preliminary or difficult to compare, and highlight practical research gaps relevant to nutrition policy and future intervention design [33]. It may also help distinguish between questions of nutritional efficacy, technical feasibility, and translational relevance, which are often conflated when fortified eggs are discussed as if they were a single intervention category.

Therefore, the objective of this review was to map and synthesize the available human intervention evidence on fortified egg consumption and its reported nutritional and health-related outcomes. Although eggs fortified with *n*-3 fatty acids, carotenoids, iodine, vitamin D, or multiple micronutrients should not be regarded as a single uniform nutritional intervention, they were reviewed together because they share a common food vehicle and nutrient-delivery platform. The aim of this review was therefore not to assume equivalence across nutrient-specific strategies, but to characterize the scope, distribution, and focus of research on eggs as a delivery vehicle. Specifically, this review sought to: (1) characterize the types of fortified egg interventions that have been studied; (2) identify the populations and outcome domains represented in the literature; and (3) summarize reported effects on nutrient exposure, nutritional status, and health-related indicators. Where reported, the review also considered information relevant to fortification procedures and nutrient deposition.

The value of this review lies not in treating all fortified eggs as nutritionally equivalent, but in clarifying how this shared food vehicle has been studied across distinct nutrient targets and in identifying where evidence remains too sparse, heterogeneous, or methodologically limited to support broader conclusions.

## 2. Materials and Methods

### 2.1. Review Design and Reporting Framework

This study was conducted to map and characterize the available human intervention evidence on fortified eggs and their reported nutritional and health-related outcomes. This approach was considered appropriate because the literature encompasses multiple fortification strategies, heterogeneous intervention designs, diverse population groups, variable comparator conditions, and a broad range of outcome measures. Accordingly, the aim of the review was not to estimate a pooled effect size, but rather to describe the extent, nature, and distribution of the available evidence and to identify key research gaps. The available evidence also consisted predominantly of short- to medium-term studies conducted in healthy adults, with relatively limited representation of vulnerable subgroups and few assessments of long-term or clinically meaningful outcomes.

The review methodology was informed by the framework originally proposed by Arksey and O’Malley and further developed in subsequent methodological guidance for evidence-mapping studies [34]. Reporting followed the Preferred Reporting Items for Systematic Reviews and Meta-Analyses extension for Scoping Reviews (PRISMA-ScR) [35]. The review protocol was prospectively registered on the Open Science Framework (OSF; 10.17605/OSF.IO/2C9GP). Consistent with this methodological approach, formal critical appraisal or risk-of-bias assessment of the included studies was not undertaken, as the primary objective was evidence mapping and characterization rather than comparative effectiveness appraisal or quantitative effect estimation.

### 2.2. Eligibility Criteria

Eligibility criteria were defined a priori using the SPIDER framework (Sample, Phenomenon of Interest, Design, Evaluation, and Research type) [36]. SPIDER was selected because it is well suited to reviews that aim to map heterogeneous intervention evidence and diverse outcome measures rather than estimate a single pooled comparative effect [37]. Studies were eligible if they: (1) involved human participants from the general population or specific subpopulations; (2) evaluated the consumption of fortified, enriched, designer, or bio-enhanced eggs intended to modify nutrient composition; (3) used a quantitative intervention design; and (4) reported at least one nutritional or health-related outcome. Nutritional outcomes included measures of nutrient exposure, bioavailability, or status (e.g., blood or urinary biomarkers), whereas health-related outcomes included physiological, metabolic, visual, vascular, inflammatory, or other clinically relevant indicators.

Studies were excluded if they were animal studies, in vitro studies, observational studies, reviews, conference abstracts without sufficient data, protocols, commentaries, or non-intervention reports. Studies evaluating egg analogs, plant-based egg substitutes, composite foods fortified with egg powder, or interventions in which eggs were not the primary fortified vehicle were also excluded. This restriction was applied to maintain a clear focus on fortified eggs as a distinct food-based delivery platform.

Because the purpose of this review was to map the breadth and structure of the available human evidence rather than to estimate a single pooled effect, different fortification categories were included within the same review framework. However, these categories were not assumed to represent a uniform nutritional intervention. Their findings were interpreted with attention to nutrient-specific biological mechanisms, target populations, and outcome domains.

### 2.3. Information Sources and Search Strategy

A comprehensive literature search was conducted in PubMed, Embase, Web of Science, the Cochrane Library, CNKI, Wanfang Data, and SinoMed. The searches were conducted on 17 June 2025 and covered studies published from 1 January 2005 to 1 June 2025 in English or Chinese. Search terms combined controlled vocabulary, including Medical Subject Headings (MeSH) where applicable, with free-text terms related to fortified eggs and intervention studies, such as “fortified egg”, “enriched egg”, “functional egg”, and “designer egg”. Boolean operators were used to combine egg-related and fortification-related concepts. The full database-specific search strategies are provided in Supplementary Materials (Section S1). To improve search completeness, the reference lists of all included studies were also screened manually for additional eligible reports.

### 2.4. Study Selection

Study selection followed PRISMA-ScR guidance [35]. Records were exported to EndNote for deduplication. Two reviewers independently screened titles and abstracts against the eligibility criteria, and potentially relevant reports were retrieved for full-text assessment. Discrepancies were resolved by discussion and, when necessary, consultation with a third reviewer. Studies excluded after full-text assessment, together with the reasons for exclusion, are provided in Supplementary Materials (Section S3).

### 2.5. Data Charting Process

Detailed characteristics and key findings of the 37 included studies, reported in 39 publications, are presented in Supplementary Materials (Section S2). One reviewer charted the data and a second reviewer independently verified the accuracy and completeness of the extracted information. Any discrepancies were resolved through discussion.

Extracted variables included author and year, country, study design, participant characteristics, fortification category, specific nutrient(s) used for fortification, comparator, intervention dose and duration, outcomes assessed, and the main findings reported by the authors. Outcomes were further charted as nutritional outcomes (e.g., biomarkers of nutrient exposure or status) or health-related outcomes (e.g., lipid, vascular, visual, inflammatory, thyroid-related, or other physiological indicators).

Information on fortification procedures, egg nutrient composition, and feed-to-egg transfer efficiency was extracted where available; however, reporting of these technical details was often incomplete or insufficiently standardized across the included studies.

### 2.6. Data Synthesis

Given the substantial heterogeneity across included studies in fortification target, nutrient dose, egg composition, intervention format, comparator, study population, duration, and outcome assessment, a quantitative synthesis was not considered appropriate. Instead, the evidence was charted descriptively and synthesized narratively. In keeping with the purpose of this review, emphasis was placed on mapping the distribution and characteristics of the evidence, identifying recurring outcome patterns within nutrient categories, and highlighting areas where the evidence base was limited, methodologically heterogeneous, or insufficient for clinically oriented interpretation.

### 2.7. Synthesis of Results

Consistent with scoping review methodology, findings were synthesized descriptively and narratively. Studies were organized according to the fortified nutrient category (e.g., *n*-3 fatty acids, carotenoids, iodine, and multiple micronutrients) and, where relevant, by population group. Outcomes were grouped into nutritional outcomes, including measures of nutrient exposure or status (e.g., serum or urinary biomarkers), and health-related outcomes (e.g., lipid profiles, thyroid-related indicators, and macular pigment optical density). Given the substantial heterogeneity in study design, intervention dose, duration, comparator conditions, and outcome assessment across the included studies, meta-analysis was not undertaken. The synthesis therefore focused on identifying patterns in the types of fortified egg interventions studied, the populations represented, the outcome domains assessed, and the direction and nature of the reported findings. The included studies were first organized according to fortification category (e.g., *n*-3 polyunsaturated fatty acids, carotenoids, iodine, vitamin D, and multi-micronutrient or antioxidant fortification). Within each category, evidence was summarized in terms of study volume, typical population, intervention duration, principal biomarker or nutritional outcomes, principal health-related outcomes, and major interpretive limitations. Findings were interpreted primarily within, rather than across, fortification categories.

## 3. Results

### 3.1. Characteristics of Included Studies

**Figure 1:**
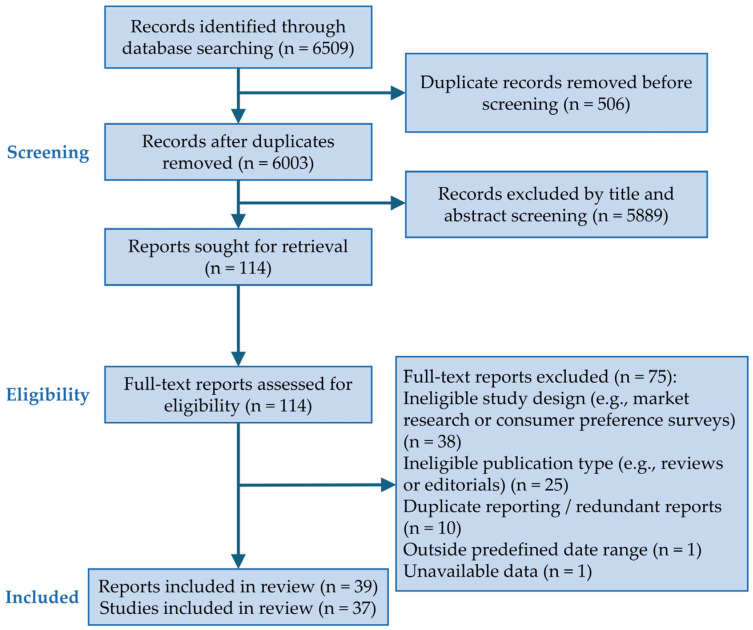
PRISMA flow diagram depicting the systematic literature search and selection process. The diagram illustrates the stages of identification, screening, eligibility, and inclusion for studies investigating the nutritional and health effects of fortified eggs on human populations. *n* represents the number of records at each stage. PRISMA, Preferred Reporting Items for Systematic Reviews and Meta-Analyses.

The database search identified 6509 records. After 506 duplicates were removed, 6003 records underwent title and abstract screening, of which 5889 were excluded. A total of 114 full-text reports were assessed for eligibility. Of these, 75 were excluded because of ineligible study design (e.g., market research or consumer preference surveys; *n* = 38), ineligible publication type (e.g., reviews or editorials; *n* = 25), duplicate reporting (*n* = 10), publication outside the predefined date range (*n* = 1), or unavailable data (*n* = 1). Ultimately, 37 unique studies, reported in 39 publications, met the inclusion criteria. The study selection process is presented in Figure 1, and the reasons for exclusion at the full-text stage are provided in Supplementary Materials (Section S3). Overall, the included evidence base was modest in size and was derived from a relatively limited number of human intervention studies, despite the broad range of fortified egg formulations identified in the literature.

### 3.2. Overview of Included Studies and Fortification Types

Table 1 summarizes the characteristics of the 37 included studies. Most studies used a parallel-group design (*n* = 28, 75.7%), whereas nine (24.3%) used a crossover design. Geographically, the evidence was concentrated in Europe (*n* = 20, 54.1%), followed by Asia (*n* = 8, 21.6%) and North America (*n* = 7, 18.9%); one study each was conducted in South America and Africa. Overall, the geographical distribution of the evidence suggests that human research on fortified eggs has been undertaken predominantly in European settings, with comparatively limited representation from other regions.

With respect to study populations, 22 studies (59.5%) enrolled generally healthy adults, including specific subgroups such as vegetarians and competitive athletes. The remaining 15 studies (40.5%) involved clinical or at-risk populations, including individuals with hypercholesterolemia, metabolic syndrome, or other cardiovascular risk factors. Notably, no eligible studies targeting pregnant women or children/infants were identified.

In terms of fortification strategies, *n*-3 fatty acid enrichment (including DHA and ALA) was the most frequently studied approach, accounting for 25 studies (67.6%). Carotenoid enrichment (including lutein and/or zeaxanthin) was the second most common category (*n* = 7, 18.9%), followed by multi-nutrient fortification (*n* = 3), iodine fortification (*n* = 1), and vitamin D fortification (*n* = 1). Intervention duration varied substantially; however, more than half of the studies were short term (≤4 weeks; *n* = 19, 51.4%), whereas only six studies (16.2%) extended beyond 12 weeks. Reported outcomes most commonly involved health-related biomarkers (e.g., lipid profiles or vascular function; *n* = 19) or a combination of nutritional and health-related indicators (*n* = 12).

Overall, the included evidence was concentrated on *n*-3 fatty acid-enriched eggs, whereas substantially fewer studies evaluated iodine, vitamin D, or other micronutrient fortification strategies. This pattern indicates that the current literature is dominated by a relatively narrow subset of fortified egg interventions, with more limited evidence available for several nutritionally relevant micronutrient-based approaches.

### 3.3. Iodine-Fortified Eggs

Only one included intervention study evaluated iodine-fortified eggs in adults, with outcomes focused on iodine intake and iodine-status biomarkers [14]. Consumption of iodine-fortified eggs increased iodine intake and urinary iodine concentration, whereas thyroid-related biomarkers remained within clinically normal ranges throughout the intervention period. No adverse events were reported. This evidence on iodine-fortified eggs was limited to a single intervention study.

### 3.4. n-3 PUFA-Enriched Eggs

The largest body of evidence concerned eggs enriched with long-chain *n*-3 PUFAs, which were evaluated in 25 studies conducted predominantly in adult populations. These studies mainly assessed changes in circulating fatty acid profiles and cardiometabolic indicators following regular consumption of *n*-3 PUFA-enriched eggs (Table 2). Taken together, this category represented the most extensively studied fortification strategy identified in the review.

Across studies, consumption of *n*-3 PUFA-enriched eggs was consistently associated with increases in DHA and, where reported, EPA concentrations in plasma, serum, or erythrocytes, indicating effective incorporation into circulating lipid pools [29,38]. Several studies also reported favorable changes in lipid-related biomarkers, most commonly reductions in triglycerides or changes in lipoprotein profiles, although the magnitude and consistency of these effects varied across studies and populations.

A smaller number of studies evaluated biomarkers of inflammation, oxidative stress, or endothelial function, and findings in these domains were mixed. Overall, the evidence for *n*-3 PUFA-enriched eggs was strongest for changes in fatty acid status biomarkers, whereas findings for broader health-related outcomes were more heterogeneous.

### 3.5. Carotenoid-Enriched Eggs

Seven studies evaluated eggs enriched with carotenoids, most commonly lutein and, in some cases, zeaxanthin. These studies primarily examined carotenoid-status biomarkers and outcomes related to visual or eye health.

Consumption of carotenoid-enriched eggs was consistently associated with increases in circulating lutein and zeaxanthin concentrations [25,39]. Several studies also reported increases in macular pigment optical density. A smaller number of studies assessed functional visual outcomes or other eye health-related indicators, with variable results.

Taken together, these studies consistently showed improvements in carotenoid-status biomarkers, whereas evidence for broader functional or clinical outcomes was more limited.

### 3.6. Eggs Fortified with Other Micronutrients

Relatively few studies evaluated eggs fortified with nutrients other than iodine, *n*-3 PUFAs, or carotenoids. Three studies investigated multi-nutrient fortified eggs, and one study evaluated vitamin D-fortified eggs.

These studies generally reported increases in circulating concentrations of the targeted nutrients. However, substantial heterogeneity in fortification approaches, study populations, and outcome measures limited direct comparison across studies. As a result, the evidence for these fortification approaches remains comparatively limited and less cohesive than that for *n*-3 PUFA- or carotenoid-enriched eggs.

### 3.7. Populations Studied and Outcomes Assessed

Most included studies were conducted in generally healthy adult populations. Fewer studies involved older adults or individuals at elevated nutritional or cardiometabolic risk. Across populations, reported outcomes were dominated by nutrient-status biomarkers, with fewer assessments of intermediate health indicators and very limited evaluation of long-term or clinically meaningful endpoints. Overall, the literature has focused primarily on short- to medium-term biomarker responses rather than long-term health outcomes [40].

### 3.8. Summary of the Evidence Landscape

This review identified an uneven evidence base, with a strong concentration of studies on eggs enriched with long-chain *n*-3 PUFAs. Far fewer studies evaluated eggs fortified with iodine, vitamin D, carotenoids, or multiple micronutrients. Across fortification types, fortified eggs were generally associated with improvements in biomarkers of the targeted nutrients, particularly fatty acid and carotenoid status, whereas broader health-related outcomes were assessed less frequently and showed greater heterogeneity.

The available evidence also consisted predominantly of short- to medium-term studies conducted in healthy adults, with relatively limited representation of vulnerable subgroups and few assessments of long-term or clinically meaningful outcomes. Overall, the current literature suggests that fortified eggs may be effective vehicles for improving specific nutrient-status biomarkers, but the evidence base remains uneven across fortification types and is still insufficient to support firm conclusions regarding longer-term clinical or public health benefits.

## 4. Discussion

This review synthesized the available human intervention evidence on fortified eggs and their reported nutritional and health-related outcomes. Across the included studies, fortified eggs were generally associated with improvements in biomarkers of the targeted nutrients, particularly for long-chain *n*-3 fatty acids and carotenoids. However, this pattern should not be interpreted as evidence of uniform effectiveness across all fortification strategies. The evidence base was concentrated in a limited number of nutrient categories, especially *n*-3-enriched eggs, whereas iodine-, vitamin D-, and multi-micronutrient-fortified eggs were represented by relatively few studies. Accordingly, the apparent consistency of nutrient-related findings reflects the distribution of the available literature as much as the strength of the underlying evidence.

A second important finding is that the evidence was much stronger for short-term nutrient delivery than for downstream health-related benefit. Many studies reported changes in circulating fatty acids, serum carotenoids, or urinary iodine, but fewer examined outcomes with clearer clinical or functional relevance, and those that did used diverse endpoints that were difficult to compare across studies. This limits the extent to which improved nutrient-status biomarkers can be taken as evidence of broader health benefit. At present, the literature supports fortified eggs more convincingly as delivery vehicles for selected nutrients than as interventions with well-established clinical effects.

The heterogeneity of the evidence base also substantially limits cross-study comparability. The included interventions differed not only in nutrient target, but also in dose, egg production method, comparator, study duration, participant characteristics, and outcome assessment. Another important limitation of the current evidence base is the relative absence of populations at greatest nutritional risk. Most studies were conducted in healthy adults, whereas pregnant women, children, older adults, and low-resource populations were largely underrepresented.

### 4.1. The Egg Matrix as a Nutrient Delivery Vehicle

A recurring finding across the included studies was that fortified eggs, particularly those enriched with long-chain *n*-3 PUFAs or carotenoids, were consistently associated with increases in circulating biomarkers of the targeted nutrients. One plausible explanation is the food matrix of the egg itself. Eggs provide lipophilic nutrients within a biologically complex matrix containing phospholipids, cholesterol, and proteins, which may facilitate micelle formation, intestinal absorption, and transport of these compounds [23,24].

Although the studies included in this review were not designed to isolate matrix-specific mechanisms directly, the observed biomarker responses should be interpreted cautiously, because few studies were designed to distinguish matrix effects from dose effects, background diet, comparator choice, or differences in baseline nutrient status. Accordingly, the current evidence suggests that eggs may function as an efficient delivery vehicle for selected lipophilic nutrients, but it does not establish that the egg matrix is uniquely or consistently superior to other food-based delivery systems. This may be particularly relevant for nutrients such as DHA, lutein, and zeaxanthin, for which bioavailability is known to depend on the surrounding food matrix and co-ingested lipids [16,24]. At the same time, the egg should not be viewed as a uniform delivery platform across all fortification strategies.

### 4.2. Fortification Process and Technical Considerations

An additional issue insufficiently addressed in much of the included literature is the technical robustness of the fortification process itself. In many studies, reporting of feed composition, fortification procedures, nutrient deposition in eggs, and feed-to-egg transfer efficiency was incomplete or inconsistent. This limits interpretation because observed human outcomes depend not only on the intervention design, but also on how reliably the target nutrients were incorporated into the eggs and maintained throughout production and consumption. Better reporting of nutrient enrichment protocols and achieved egg nutrient composition would improve reproducibility and strengthen the interpretability of future trials.

### 4.3. Research Focus and Public Health Relevance

Another notable finding was the uneven distribution of the evidence base. More than two-thirds of the included studies focused on *n*-3 PUFA-enriched eggs, whereas relatively few studies evaluated eggs fortified with iodine, vitamin D, or multiple micronutrients.

However, from a public health perspective, the relative scarcity of studies on iodine- and vitamin D-fortified eggs is notable. These nutrients remain important in the context of global micronutrient inadequacy, and the small number of available studies identified in this review reported favorable changes in nutrient-status biomarkers [14,22]. Nevertheless, these findings should be interpreted cautiously. The current available evidence is limited in volume and is derived predominantly from short- to medium-term trials in generally healthy adults, rather than from populations most vulnerable to micronutrient deficiencies.

As a result, the present evidence is not sufficient to support broad public health recommendations. Importantly, eggs enriched with *n*-3 PUFAs, iodine, carotenoids, vitamin D, or multiple nutrients represent related but distinct nutritional strategies that share a delivery platform, rather than a single intervention with uniform biological mechanisms, target populations, or health implications.

### 4.4. From Nutrient Biomarkers to Health Outcomes

A central observation from this review is that improvements in nutrient-status biomarkers did not consistently translate into broader health-related effects. In studies of *n*-3 PUFA-enriched eggs, increases in DHA and EPA status were commonly reported, and some studies observed favorable changes in triglycerides or lipoprotein profiles [23,41]. However, findings for other outcomes, including inflammation, endothelial function, or blood pressure, were less consistent [42,43]. This disconnect is important because it suggests that the field has generated more evidence for biochemical uptake than for clinically meaningful benefit.

Several factors may contribute to this heterogeneity. These include variation in study duration, baseline nutritional status, participant health status, dose of the fortified nutrient, comparator choice, and the selection of outcome measures. It is also possible that biomarker responses occur earlier or more consistently than measurable changes in clinical or functional endpoints, particularly in short-term studies. As a result, the current evidence supports the nutritional efficacy of fortified eggs more clearly than their broader clinical effectiveness.

### 4.5. Implications for Research and Practice

The findings of this review suggest several priorities for future research. First, more studies are needed on underrepresented fortification targets, particularly iodine, vitamin D, and multi-micronutrient formulations. Second, future trials should extend beyond short-term biomarker responses and incorporate longer follow-up periods, more diverse population groups, and outcomes with greater clinical or functional relevance. Third, more standardized reporting of egg nutrient composition, fortification procedures, intervention dose, and feed-to-egg transfer efficiency would improve comparability across studies and support future evidence synthesis. Fourth, greater attention should be given to implementation-relevant dimensions, including acceptability, stability, affordability, and regulatory context. Without these improvements, the literature will remain difficult to interpret and prone to overstating consistency where only partial comparability exists.

### 4.6. Strengths and Limitations

To our knowledge, this is the first scoping review to map the human intervention literature on fortified eggs across multiple nutrient fortification strategies. By following a prespecified protocol and reporting in accordance with PRISMA-ScR, this review provides a structured overview of the scope and distribution of the available evidence.

Several limitations should also be acknowledged. First, the review was restricted to studies published in English or Chinese, which may have led to the omission of relevant reports from other regions. Second, the included studies were heterogeneous with respect to fortification strategy, nutrient dose, study population, comparator, duration, and outcome assessment. This limited direct comparability across studies. Third, the evidence base was relatively small for several fortification categories, particularly iodine-, vitamin D-, and multi-micronutrient-fortified eggs, which constrained the depth of interpretation and reduced the robustness of category-specific conclusions. Fourth, reporting of fortification procedures, nutrient deposition, and transfer efficiency was often incomplete, limiting assessment of the technical consistency and reproducibility of the interventions.

Finally, because these interventions differ in biological rationale, intended target populations, and expected outcomes, cross-category synthesis should be interpreted cautiously. As this review was designed to map the evidence rather than to estimate pooled effects, formal quantitative synthesis and risk-of-bias appraisal were not undertaken, which further limits the extent to which strength of evidence or effect certainty can be inferred. Accordingly, the review is better suited to identifying patterns and research gaps than to drawing definitive conclusions about effect magnitude.

## 5. Conclusions

In summary, the available human intervention evidence indicates that fortified eggs can improve biomarkers of targeted nutrient status, particularly for long-chain *n*-3 fatty acids and carotenoids. However, the evidence is substantially stronger for short-term biochemical response than for clearly established clinical or public health benefit, and it remains unevenly distributed across fortification categories.

The current literature is dominated by short- to medium-term studies in generally healthy adults, with comparatively little evidence on iodine, vitamin D, and other micronutrient fortification strategies, or populations at greater nutritional risk. Overall, the available evidence more consistently supports fortified eggs as vehicles for short-term nutrient delivery than as interventions with clearly established clinical or public health benefit. Further well-designed studies, with clearer reporting of fortification methods, are needed to clarify the role of fortified eggs in nutritional practice and public health strategies.

## Figures and Tables

**Table 1:** Characteristics of the 37 Included Studies.

Characteristic	Category	Number of Studies	Percentage (%)
Study design (RCTs)	Parallel Group	28	75.7%
	Crossover	9	24.3%
Target population	Healthy adults (incl. athletes/vegetarians)	22	59.5%
	Clinical patients/at-risk individuals ^1^	15	40.5%
Geographical region	Europe	20	54.1%
	Asia	8	21.6%
	North America	7	18.9%
	South America	1	2.7%
	Africa	1	2.7%
Type of egg fortification	*n*-3 fatty acids (including PUFA and DHA)	25	67.6%
	Carotenoids (lutein/zeaxanthin)	7	18.9%
	Multiple micronutrients	3	8.1%
	Iodine	1	2.7%
	Vitamin D	1	2.7%
Intervention duration	Short-term (≤4 weeks)	19	51.4%
	Medium-term (5–12 weeks)	12	32.4%
	Long-term (>12 weeks)	6	16.2%
Outcome category reported	Health-related outcomes only	19	51.4%
	Nutritional biomarkers only	6	16.2%
	Both nutritional and health-related outcomes	12	32.4%

^1^ The “Clinical patients/at-risk individuals” category includes participants with hypercholesterolemia, metabolic syndrome (MetS), cardiovascular disease (CVD) risk factors, iodine deficiency, or early signs of age-related macular degeneration (AMD). The “Healthy adults” category includes general healthy populations, vegetarians, and competitive athletes. Abbreviations: RCT: randomized controlled trial; *n*-3 PUFA: omega-3 polyunsaturated fatty acids; DHA: docosahexaenoic acid; CVD: cardiovascular disease; MetS: metabolic syndrome; AMD: age-related macular degeneration.

**Table 2:** Category-level evidence landscape of fortified egg intervention studies included in the review.

Fortification Category	No. of Studies	Typical Population	Typical Duration	Main Biomarker/ Nutritional Outcomes	Main Health-Related Outcomes	Key Limitations/ Interpretive Cautions
*n*-3 PUFA-enriched eggs	25	Mostly healthy adults; some athletes, vegetarians, and participants with metabolic syndrome, hyperlipidemia, or higher cardiometabolic risk.	Mainly 3–8 weeks; fewer studies lasted 3–6 months; one acute postprandial trial.	Consistent rises in DHA/EPA, RBC omega-3 index, and fatty acid status; some studies also reported lower inflammatory markers or altered glycosylation/leukocyte activation.	Most consistent findings were lower triglycerides and better HDL-C or vascular reactivity. Effects on LDL-C, glucose, insulin sensitivity, blood pressure, and broader cardiometabolic outcomes were mixed.	Largest evidence base, but still dominated by short-term adult studies. Dose, comparators, co-interventions, and endpoints varied widely, limiting cross-study comparability and inference on long-term clinical outcomes.
Carotenoid-enriched eggs	7	Mainly healthy adults, including low-MPOD groups and adults with early age-related macular changes.	Mostly 8–12 weeks; one study lasted 6 months and one 1 year.	Consistent increases in serum/plasma lutein and/or zeaxanthin; in several studies bioavailability was comparable to supplements.	MPOD often increased or trended upward, but effects on visual function, contrast sensitivity, and metabolic markers were less consistent. Lipid safety findings were generally neutral.	Smaller and more outcome-specific evidence base than for *n*-3 eggs, with eye-health endpoints dominating. Functional benefits were less consistent than biomarker responses, and some studies used mixed nutrient enrichment or different food formats.
Iodine-enriched eggs	1	Iodine-deficient women.	5 days.	Rapid increase in urinary iodine concentration from deficient to adequate ranges.	Evidence mainly supports short-term correction of iodine status; no clear broader health endpoint evidence was available.	Single-study evidence base with very short follow-up. Conclusions should be restricted to short-term biomarker efficacy rather than broader clinical benefit.
Vitamin D-fortified egg interventions	1	Women of Danish and Pakistani origin during winter.	12 weeks.	Maintenance of serum 25(OH)D and lower prevalence of severe winter deficiency.	Findings support prevention of seasonal decline in vitamin D status, but broader health effects were not robustly established.	Single-study evidence base; the intervention also included other vitamin D-fortified foods, so attribution to eggs alone is limited and needs replication.
Multi-micronutrient/antioxidant-fortified eggs	3	Healthy young adults, generally healthy adults, and adults with elevated cardiovascular risk.	Mostly 12 weeks, with one 21-day trial.	Mixed biomarker findings, including lower oxidative stress or inflammatory markers in some studies and higher circulating carotenoids in others.	Some studies suggested better endothelial or microvascular responses, whereas others found no clear advantage over regular eggs for visual or lipid outcomes.	Very small and heterogeneous evidence base. Because several products combined multiple nutrients, attribution to any single component is difficult; populations, formulations, and endpoints also differed substantially.

Abbreviations: 25(OH)D, 25-hydroxyvitamin D; DHA, docosahexaenoic acid; EPA, eicosapentaenoic acid; HDL-C, high-density lipoprotein cholesterol; LDL-C, low-density lipoprotein cholesterol; MPOD, macular pigment optical density; *n*-3 PUFA, omega-3 polyunsaturated fatty acids; RBC, red blood cell.

## Data Availability

No new data were created in this study. Data sharing is not applicable to this article.

## Supplementary Materials

The following supporting information can be downloaded at: https://www.mdpi.com/article/10.3390/nu18132189/s1, Section S1: Full Database-specific Search Strategies; Section S2: Characteristics of Included Studies (*n* = 37); Section S3: List of Excluded Studies at Full-text Screening; Section S4: PRISMA-ScR Checklist.

## Abbreviations

The following abbreviations are used in this manuscript:

25(OH)D	25-hydroxyvitamin D
ALA	alpha-linolenic acid
AMD	age-related macular degeneration
CNKI	China National Knowledge Infrastructure
CVD	cardiovascular disease
DHA	docosahexaenoic acid
EPA	eicosapentaenoic acid
HDL-C	high-density lipoprotein cholesterol
LDL-C	low-density lipoprotein cholesterol
MeSH	Medical Subject Headings
MetS	metabolic syndrome
MPOD	macular pigment optical density
*n*-3 PUFA	omega-3 polyunsaturated fatty acid
OSF	Open Science Framework
PRISMA-ScR	Preferred Reporting Items for Systematic Reviews and Meta-Analyses extension for Scoping Reviews
PUFA	polyunsaturated fatty acid
RBC	red blood cell
RCT	randomized controlled trial
ROS	reactive oxygen species
SPIDER	Sample, Phenomenon of Interest, Design, Evaluation, and Research type
