# Supplementary material for: Fortified Eggs as Food-Based Vehicles for Nutrient Delivery: A Scoping Review of Human Intervention Studies
Source: Nutrients. 2026 Jul 5;18(13):2189. doi: 10.3390/nu18132189 (PMC13364057; doi:10.3390/nu18132189)
Supplement: Supplementary file 1 [file nutrients-18-02189-s001.zip › nutrients-4370623-supplementary.pdf]

Supplementary Materials

Manuscript Title:

Fortified Eggs as Food-Based Vehicles for Nutrient Delivery: A Scoping Review of Human Intervention Studies

Authors:

Liusen Wang[1,2], Hongru Jiang[1,2], Weiyi Li[1,2], Lixin Hao[1,2], Ziyan Liu[3], Xu Yan[3], Jingming Yang[1,2], Yang Liu[1,2] and Chao Gao[1,2,*]

Correspondence:

gaochao@ninh.chinacdc.cn (C.G.)

National Institute for Nutrition and Health, Chinese Center for Disease Control and Prevention, Beijing, China.

Table of Contents

Item	Description	Page No.
**Section S1**	**Full Database-specific Search Strategies**<br>Detailed search strings for PubMed, Embase, Web of Science, Cochrane Library, CNKI, Wanfang Data, and SinoMed.	1
**Section S2**	**Characteristics of Included Studies (n = 37)**<br>Comprehensive data extraction table including study design, population details, intervention protocols, and key outcomes for all 37 included trials.	8
**Section S3**	**List of Excluded Studies at Full-text Screening**<br>Bibliographic details of the 75 studies excluded after full-text review, with specific reasons for exclusion (e.g., ineligible design, wrong publication type).	29
**Section S4**	**PRISMA-ScR Checklist**<br>Preferred Reporting Items for Systematic reviews and Meta-Analyses extension for Scoping Reviews (PRISMA-ScR) checklist.	35

Section S1: Full Database-specific Search Strategies

Search date: June 17, 2025

Time period covered: January 1, 2005 – June 1, 2025

1. PubMed

#1 "Food, Fortified"[MeSH Terms] OR "Biofortification"[MeSH Terms] OR "foods fortified"[Title/Abstract] OR "fortified food*"[Title/Abstract] OR "food* supplemented"[Title/Abstract] OR "supplemented food*"[Title/Abstract] OR "enriched food*"[Title/Abstract] OR "food* enriched"[Title/Abstract] OR "biofortified crop*"[Title/Abstract] OR "crop* biofortified"[Title/Abstract]

#2 "Eggs"[MeSH Terms]

#3 "Biofortified egg*"[Title/Abstract] OR "Fortified egg*"[Title/Abstract] OR "Enriched egg*"[Title/Abstract] OR "Bioenhanced egg*"[Title/Abstract] OR "Functional egg*"[Title/Abstract] OR "Nutritionally Fortified egg*"[Title/Abstract] OR "Functional Health egg*"[Title/Abstract] OR "Health-Promoting egg*"[Title/Abstract] OR "Functional Poultry egg*"[Title/Abstract] OR "Specialty egg*"[Title/Abstract]

#4 (#1 AND #2) OR #3

#5 #4 AND ("2005/01/01"[Date - Publication] : "2025/06/01"[Date - Publication])

#6 #5 AND Humans[MeSH Terms]

2. Embase (Ovid / Embase.com)

#1 'egg'/exp OR 'egg'

#2 'biofortification'/exp OR 'fortified food'/exp

#3 egg*:ti,ab,kw

#4 'food*, fortified':ti,ab,kw OR 'fortified food*':ti,ab,kw OR 'food*, supplemented':ti,ab,kw OR 'supplemented food*':ti,ab,kw OR 'enriched

food*':ti,ab,kw OR 'food*, enriched':ti,ab,kw OR 'biofortified crop*':ti,ab,kw OR 'crop*, biofortified':ti,ab,kw

#5 'biofortified egg*':ti,ab,kw OR 'fortified egg*':ti,ab,kw OR 'enriched egg*':ti,ab,kw OR 'bioenhanced egg*':ti,ab,kw OR 'functional egg*':ti,ab,kw OR 'nutritionally fortified egg*':ti,ab,kw OR 'functional health egg*':ti,ab,kw OR 'health-promoting egg*':ti,ab,kw OR 'functional poultry egg*':ti,ab,kw OR 'specialty egg*':ti,ab,kw

#6 #1 OR #3

#7 #2 OR #4

#8 (#6 AND #7) OR #5

#9 #8 AND [humans]/lim AND [embase]/lim AND [2005-2025]/py

3. Web of Science (Core Collection)

Set Query

#1 TS=(Egg*)

#2 TS=("Food, Fortified") OR TS=(Biofortification) OR ALL=("Food*, Fortified") OR ALL=("Fortified Food*") OR ALL=("Food*, Supplemented") OR ALL=("Supplemented Food*") OR ALL=("Enriched Food*") OR ALL=("Food*, Enriched") OR ALL=("Biofortified Crop*") OR ALL=("Crop*, Biofortified")

#3 TI=("biofortified egg*") OR TI=("fortified egg*") OR TI=("enriched egg*") OR TI=("bioenhanced egg*") OR TI=("functional egg*") OR TI=("nutritionally fortified egg*") OR TI=("functional health egg*") OR TI=("health-promoting egg*") OR TI=("functional poultry egg*") OR TI=("specialty egg*")

#4 #1 AND #2

#5 #4 OR #3

#6 TI=("rat?" OR "mouse" OR "mice" OR "pig?" OR "murine" OR "swine" OR "porcine" OR "piglets" OR "sheep" OR "lamb?" OR "rabbit?" OR "cat?" OR "dog?" OR "cattle" OR "bovine" OR "monkey?" OR "marmoset")

#7 #5 NOT #6

Note Timespan: 2005-01-01 to 2025-06-01

4. Cochrane Library

ID Search

#1 MeSH descriptor: [Eggs] explode all trees

#2 (egg*):ti,ab,kw

#3 MeSH descriptor: [Food, Fortified] explode all trees

#4 MeSH descriptor: [Biofortification] explode all trees

#5 (Food*, Fortified):ti,ab,kw OR (Fortified Food*):ti,ab,kw OR (Food*, Supplemented):ti,ab,kw OR (Supplemented Food*):ti,ab,kw OR (Enriched Food*):ti,ab,kw

#6 (Food*, Enriched):ti,ab,kw OR (Biofortified Crop*):ti,ab,kw OR (Crop*, Biofortified):ti,ab,kw

#7 rat?:ti OR mouse:ti OR mice:ti OR pig?:ti OR murine:ti OR swine:ti OR porcine:ti OR piglets:ti OR sheep:ti OR lamb?:ti OR rabbit?:ti OR cat?:ti OR dog?:ti OR cattle:ti OR bovine:ti OR monkey?:ti OR marmoset:ti

#8 (Biofortified egg*):ti,ab,kw OR (Fortified egg*):ti,ab,kw OR (Enriched egg*):ti,ab,kw OR (Bioenhanced egg*):ti,ab,kw OR (Functional egg*):ti,ab,kw

#9 (Nutritionally Fortified egg*):ti,ab,kw OR (Functional Health egg*):ti,ab,kw OR (Health-Promoting egg*):ti,ab,kw OR (Functional Poultry egg*):ti,ab,kw OR (Specialty egg*):ti,ab,kw

#10 #1 OR #2

#11 #3 OR #4 OR #5 OR #6

#12 (#10 AND #11) OR #8 OR #9

#13 #12 NOT #7

Note Publication date Between Jan 2005 and Jun 2025

5. SinoMed (CBM - China Biology Medicine disc)

No.	Search Strategy (Chinese terms [English translation])
#1	(“鸡蛋” [Chicken egg] OR “禽蛋” [Poultry egg] OR “蛋” [Egg])
#2	(“强化” [Fortified] OR “生物强化” [Biofortified] OR “富集” [Enriched] OR “功能性” [Functional])
#3	(“功能蛋” [Functional egg] OR “强化蛋” [Fortified egg] OR “富营养蛋” [Nutrient-enriched egg] OR “保健蛋” [Health-care egg] OR “富硒蛋” [Selenium-rich egg] OR “富碘蛋” [Iodine-rich egg] OR “高锌蛋” [High-zinc egg] OR “DHA 蛋” [DHA egg])
#4	(#1 AND #2) OR #3
#5	#4 AND “人类” [Humans] AND 2005-2025 [Date]

6. CNKI (China National Knowledge Infrastructure)

No.	Search Strategy (Chinese terms [English translation])
#1	Subject/Title/Abstract = (“鸡蛋” [Chicken egg] OR “禽蛋” [Poultry egg])
#2	Subject/Title/Abstract = (“强化” [Fortification] OR “生物强化” [Biofortification] OR “富集” [Enrichment] OR “添加” [Supplemented])
#3	Subject/Title/Abstract = (“功能蛋” [Functional egg] OR “营养蛋”

No.	Search Strategy (Chinese terms [English translation])
	[Nutritional egg] OR “富硒” [Selenium-rich] OR “富碘” [Iodine-rich] OR “富锌” [Zinc-rich] OR “欧米伽-3” [Omega-3] OR “DHA”)
#4	(#1 AND #2) OR (#1 AND #3)
Note	Date range: 2005-01-01 to 2025-06-01; Literature Type: Journal Articles & Dissertations.

7. Wanfang Data

No.	Search Strategy (Chinese terms [English translation])
#1	(Title/Keywords:(“鸡蛋” [Chicken egg]) OR Title/Keywords:(“禽蛋” [Poultry egg]))
#2	(Title/Keywords:(“强化” [Fortified]) OR Title/Keywords:(“生物强化” [Biofortified]) OR Title/Keywords:(“富集” [Enriched]) OR Title/Keywords:(“功能性” [Functional]))
#3	(Title/Keywords:(“功能蛋” [Functional egg]) OR Title/Keywords:(“富硒蛋” [Selenium-rich egg]) OR Title/Keywords:(“富碘蛋” [Iodine-rich egg]) OR Title/Keywords:(“DHA 蛋” [DHA egg]))

No.	Search Strategy (Chinese terms [English translation])
#4	(#1 AND #2) OR #3
Note	Date range: 2005-01-01 to 2025-06-01.

Section S2: Characteristics of Included Studies

Table S1. Detailed characteristics of the randomized controlled trials included in the scoping review.

Reference (Author, Year) & Location	Study Design	Population (n, Health Status)	Intervention vs. Comparison (Daily Dose)	Duration	Major Outcomes Assessed	Key Findings
**Iodine Fortification**						
(Charoensiriwatana et al. 2010) (Thailand)	Pre-post-trial	n=124; Iodine deficient women	Iodine-enriched vs. Regular eggs (1 egg/day)	5 days	Urinary iodine (UIC)	**Effective.** Median UIC increased from deficiency (6.87 µg/dL) to optimal levels (13.09 µg/dL).
**n-3 PUFA Fortification**						
(Njembe et al. 2021) (Belgium)	RCT, Parallel, Double-blind	n=24; MetS risk factors	n-3 PUFA vs. Oleic acid eggs (2 eggs/day)	3 months	Waist circumference, Lipids	**Effective.** Waist circumference significantly decreased; HDL-C increased; no change in glucose.

Table S1. Detailed characteristics of the randomized controlled trials included in the scoping review.

Reference (Author, Year) & Location	Study Design	Population (n, Health Status)	Intervention vs. Comparison (Daily Dose)	Duration	Major Outcomes Assessed	Key Findings
(Azgar et al. 2024) (USA)	RCT, Crossover	n=31; Healthy	Bio-enhanced n-3 egg vs. Chicken vs. Supp (Acute dose)	Acute (8h)	Plasma DHA availability	**Effective.** Bio-enhanced eggs significantly increased plasma DHA compared to enriched poultry.
(Gillingham et al. 2005) (Canada)	RCT, Crossover, Double-blind	n=15; Hypercholesterolemic men	DHA-enriched vs. Control eggs (2 eggs/day)	21 days	Lipids, Fatty acid profile	**Effective.** Increased serum DHA (+22%) and total n-3 PUFA with no adverse effect on TC or LDL-C ratios.
(Radanović et al. 2023) (Croatia)	RCT, Parallel, Double-blind	n=20; Healthy young adults	n-3 PUFA vs. Regular eggs (3 eggs/day)	21 days	Fatty acid profile, Blood count	**Effective.** Improved serum n-6/n-3 ratio (-27%) and decreased SFA; no negative metabolic effects.

Table S1. Detailed characteristics of the randomized controlled trials included in the scoping review.

Reference (Author, Year) & Location	Study Design	Population (n, Health Status)	Intervention vs. Comparison (Daily Dose)	Duration	Major Outcomes Assessed	Key Findings
(Kolar et al. 2023) (Croatia)	RCT, Parallel, Double-blind	n=31; Male athletes	Nutri4 (n-3, Se, Vit E, Lut) vs. Regular eggs (3 eggs/day)	21 days	Microvascular function, Oxidative stress	**Effective.** Improved microvascular adaptation to stress and reduced ROS; no change in lipids.
(Stupin et al. 2021) (Croatia)	RCT, Parallel, Double-blind	n=54; Healthy & CVD patients	n-3 PUFA vs. Regular eggs (3 eggs/day)	21 days	IgG/Plasma protein N-glycosylation	**Effective.** Shifted glycosylation profiles towards less inflammatory patterns in both cohorts.
(Schnebelen-Berthier et al. 2021) (France)	RCT, Parallel, Double-blind	n=98; Healthy	Lutein/DHA vs. Standard eggs (2 eggs/day)	4 months	MPOD, Plasma DHA/Lutein	**Mixed.** Plasma DHA and lutein increased significantly; MPOD increased in both groups with no significant group difference.

Table S1. Detailed characteristics of the randomized controlled trials included in the scoping review.

Reference (Author, Year) & Location	Study Design	Population (n, Health Status)	Intervention vs. Comparison (Daily Dose)	Duration	Major Outcomes Assessed	Key Findings
(Kolar et al. 2021) (Croatia)	RCT, Parallel, Double-blind	n=23; Athletes	n-3 PUFA vs. Regular eggs (3 eggs/day)	21 days	Microvascular function	**Effective.** Enhanced resting microvascular function and reactivity to exercise stress.
(Stupin et al. 2020) (Croatia)	RCT, Parallel, Double-blind	n=40; Healthy young adults	n-3 PUFA vs. Regular eggs (3 eggs/day)	21 days	Microvascular function, Inflammation	**Effective.** Improved endothelial vasodilation; reduced pro-inflammatory INF-γ and increased anti-inflammatory IL-10.
(Shakoor et al. 2020) (Pakistan)	RCT, Crossover, Single-blind	n=21; Metabolic Syndrome	n-3 eggs vs. Regular eggs vs. No eggs (2 eggs/day)	5 weeks	Lipids, BP, Insulin	**Effective.** Reduced triglycerides, SBP/DBP, and improved insulin sensitivity compared to controls.

Table S1. Detailed characteristics of the randomized controlled trials included in the scoping review.

Reference (Author, Year) & Location	Study Design	Population (n, Health Status)	Intervention vs. Comparison (Daily Dose)	Duration	Major Outcomes Assessed	Key Findings
(Mihalj et al. 2020) (Croatia)	RCT, Parallel, Double-blind	n=20; Healthy young adults	n-3 PUFA vs. Regular eggs (2 eggs/day)	3 weeks	Leukocyte activation, Oxidative stress	**Effective.** Reduced leukocyte activation markers (CD11a); increased SOD activity.
(Jaček et al. 2020) (Czech Rep)	RCT, Parallel, Single-blind	n=28; Healthy men	n-3 enriched chicken+eggs vs. Control (1 egg + meat/day)	8 weeks	RBC fatty acids, Lipids	**Mixed.** Increased RBC n-3 content and Omega-3 Index; no significant changes in lipids or inflammatory markers.
(Stupin et al. 2018) (Croatia)	RCT, Parallel, Double-blind	n=36; Healthy young adults	n-3 PUFA vs. Regular eggs (3 eggs/day)	3 weeks	Microvascular function, BP, TG	**Effective.** Improved microvascular reactivity; significantly reduced BP, triglycerides, and hsCRP.

Table S1. Detailed characteristics of the randomized controlled trials included in the scoping review.

Reference (Author, Year) & Location	Study Design	Population (n, Health Status)	Intervention vs. Comparison (Daily Dose)	Duration	Major Outcomes Assessed	Key Findings
(Stanton et al. 2020)(Ireland)	RCT, 2x2 Factorial, Double-blind	n=161; Healthy	n-3 enriched chicken/eggs (~3 eggs/week)	6 months	Omega-3 Index, BP	**Effective.** Significant increase in Omega-3 Index; lowered diastolic BP and heart rate.
(West et al. 2014) (USA)	RCT, Crossover, Single-blind	n=15; Vegetarian women	n-3 eggs vs. Regular eggs vs. Walnuts (6 eggs/week)	8 weeks	Choline metabolites	**Effective.** n-3 eggs increased plasma free choline and betaine compared to no-egg control; TMAO was unaffected.
(Imran et al. 2014) (Pakistan)	RCT, Crossover	n=24; Hypercholesterolemic	Omega-3 vs. Regular eggs (6 eggs/week)	3 weeks	Lipid profile	**Effective.** Significantly increased HDL-C (+10.3%) and decreased triglycerides (-14.2%) without raising TC.

Table S1. Detailed characteristics of the randomized controlled trials included in the scoping review.

Reference (Author, Year) & Location	Study Design	Population (n, Health Status)	Intervention vs. Comparison (Daily Dose)	Duration	Major Outcomes Assessed	Key Findings
(Burns-Whitmore et al. 2010) (USA)	RCT, Crossover, Single-blind	n=26; Vegetarians	n-3 eggs vs. Organic eggs vs. No eggs (6 eggs/week)	8 weeks	Serum lutein/zeaxanthin	**Effective.** Both egg types significantly increased serum lutein compared to no-egg control.
(Ohman et al. 2008) (Sweden)	RCT, Crossover, Double-blind	n=19; Healthy >45y	n-3 PUFA vs. Standard eggs (1 egg/day)	1 month	ApoB/ApoA1, Glucose	**Effective.** n-3 eggs increased ApoA1, decreased ApoB/ApoA1 ratio and plasma glucose.
(Bovet et al. 2007) (Seychelles)	RCT, Crossover, Double-blind	n=25; Healthy	Fish-oil enriched vs. Regular eggs (5 eggs/week)	3 weeks	Serum triglycerides	**Effective.** Serum triglycerides decreased by 16-18%; no change in other cholesterol fractions.

Table S1. Detailed characteristics of the randomized controlled trials included in the scoping review.

Reference (Author, Year) & Location	Study Design	Population (n, Health Status)	Intervention vs. Comparison (Daily Dose)	Duration	Major Outcomes Assessed	Key Findings
(Fakhrzadeh et al. 2005) (Iran)	RCT, Parallel, Double-blind	n=42; Healthy	n-3 PUFA vs. Standard eggs (2 eggs/day)	6 weeks	Insulin, hsCRP	**Effective.** Significant reduction in fasting insulin and hsCRP in the fortified egg group.
(Kolobarić et al. 2021) (Croatia)	RCT (Abstract)	n=40; Healthy	n-3 PUFA vs. Regular eggs (Dose NR)	3 weeks	E-series Resolvins	**Effective.** Increased serum Resolvin E1 and decreased PGE2/PGE3 ratio (anti-inflammatory shift).
(Hegde et al. 2011) (India)	RCT (Abstract)	n=49; Healthy	n-3 enriched eggs (8/week) vs. Fish oil capsules	2 weeks	Lipid profile	**Effective.** n-3 eggs improved TG and TC levels similarly to fish oil capsules.
(Drenjancevic et al. 2022) (Croatia)	RCT (Abstract)	n=130; Mixed (Healthy/CVD)	n-3 PUFA vs. Regular eggs (3 eggs/day)	3 weeks	Microvascular function, Inflammation	**Mixed.** Improved vascular flow in healthy adults but not

Table S1. Detailed characteristics of the randomized controlled trials included in the scoping review.

Reference (Author, Year) & Location	Study Design	Population (n, Health Status)	Intervention vs. Comparison (Daily Dose)	Duration	Major Outcomes Assessed	Key Findings
						CVD patients; reduced inflammation markers in CVD group.
(Rasic et al. 2015) (Croatia)	RCT (Abstract)	n=20; Healthy men	Omega-3 vs. Regular eggs (3 eggs/day)	3 weeks	Oxidative stress (TBARS)	**Effective.** Significant reduction in lipid peroxidation (TBARS) in the omega-3 group.
(Burns-Whitmore et al. 2014) (USA)	RCT, Crossover, Single-blind	n=20; Vegetarians	n-3 eggs vs. Regular eggs vs. Walnuts (6 eggs/week)	8 weeks	CVD risk markers	**Mixed.** Walnuts were superior for lipids (TC/LDL); n-3 eggs effectively increased RBC DHA and significantly reduced CRP compared to walnuts.

Table S1. Detailed characteristics of the randomized controlled trials included in the scoping review.

Reference (Author, Year) & Location	Study Design	Population (n, Health Status)	Intervention vs. Comparison (Daily Dose)	Duration	Major Outcomes Assessed	Key Findings
**Carotenoid Fortification**						
(Galvis et al. 2023) (Colombia)	RCT, Parallel	n=105; Healthy	Annatto vs. Regular eggs vs. Egg whites (2 eggs/day)	8 weeks	Lipids, Inflammation	**Null.** No significant changes in lipids, apolipoproteins, or inflammation; confirmed safety.
(Kelly et al. 2017) (Ireland)	RCT, Parallel, Single-blind	n=50; Healthy	Lutein/Zeaxanthin vs. Regular eggs (1 egg/day)	8 weeks	MPOD, Serum carotenoids	**Mixed.** Serum carotenoids increased significantly; MPOD and visual function did not change significantly in 8 weeks.
(Lu et al. 2024) (China)	RCT, Parallel,	n=100; Healthy	Carotenoid-enriched (Red palm	6 months	Fatty acid profile, Microbiota	**Effective.** Decreased serum SFA, increased

Table S1. Detailed characteristics of the randomized controlled trials included in the scoping review.

Reference (Author, Year) & Location	Study Design	Population (n, Health Status)	Intervention vs. Comparison (Daily Dose)	Duration	Major Outcomes Assessed	Key Findings
	Double-blind		oil) vs. Regular eggs (1 egg/day)			PUFA; positively modulated gut microbiota diversity.
(van der Made et al. 2014) (Netherlands)	RCT, Parallel, Double-blind	n=101; Early AMD signs	Drink with Lutein-enriched yolk vs. Control (1.5 yolks/day)	1 year	Plasma lutein, Lipids	**Effective.** Plasma lutein increased (+83%); no adverse effects on serum lipids or lipoproteins.
(Kelly et al. 2014) (Netherlands)	RCT, Parallel, Double-blind	n=100; Healthy (Low MPOD)	Lutein-egg vs. Zeaxanthin-egg vs. Control (1 egg/day)	90 days	MPOD, Serum carotenoids	**Mixed.** Serum lutein/zeaxanthin increased significantly (equivalent to supplements); MPOD changes were not significant.

Table S1. Detailed characteristics of the randomized controlled trials included in the scoping review.

Reference (Author, Year) & Location	Study Design	Population (n, Health Status)	Intervention vs. Comparison (Daily Dose)	Duration	Major Outcomes Assessed	Key Findings
(Adabi et al. 2010) (Iran)	RCT, Parallel	n=16; Healthy men	Lutein-enriched (High dose) vs. Control eggs (1 egg/day)	4 weeks	Plasma lutein	**Effective.** Highest dosage group showed significant increase in plasma lutein.
(Ata et al. 2010); (Ata, Barona, and Fernández 2011) (USA)	RCT (Abstract)	n=33-34; Healthy	Lutein-enriched vs. Regular eggs vs. Substitute (1 egg/day)	12 weeks	Lipid profile (HDL, LDL, ApoB), MPOD, Plasma lutein, CRP, Insulin, HOMA-IR	**Effective.** Both egg groups: Increased HDL-C, MPOD, and plasma lutein; Decreased ApoB and CRP. (2010) Lutein-enriched egg only: Significantly reduced plasma insulin and HOMA-IR compared to baseline/control. (2011)

Table S1. Detailed characteristics of the randomized controlled trials included in the scoping review.

Reference (Author, Year) & Location	Study Design	Population (n, Health Status)	Intervention vs. Comparison (Daily Dose)	Duration	Major Outcomes Assessed	Key Findings
**Vitamin D Fortification**						
(Grønborg et al. 2020) (Denmark)	RCT, Double-blind, Stratified	n=143; Danish/Pakistani women	Vit D fortified egg/dairy vs. Control (~1 egg/day + others)	12 weeks	Serum 25(OH)D	**Effective.** Prevented winter decline in Vit D and reduced prevalence of severe deficiency.
**Multi-Nutrient / Combined Fortification**						
(Hu et al. 2024) (Singapore)	RCT, Double-blind	n=40; Healthy	Antioxidant-enriched vs. Regular eggs (2 eggs/day)	12 weeks	Visual function, MPOD	**Null/Mixed.** Visual function improved in both groups; no specific advantage of fortification over regular eggs found.

Table S1. Detailed characteristics of the randomized controlled trials included in the scoping review.

Reference (Author, Year) & Location	Study Design	Population (n, Health Status)	Intervention vs. Comparison (Daily Dose)	Duration	Major Outcomes Assessed	Key Findings
(Šušnjara et al. 2022); (Šušnjara et al. 2023) (Croatia)	RCT, Parallel, Double-blind	n=34; Healthy young adults	Nutri4 (n-3, Se, Vit E, Lut) vs. Regular eggs (3 eggs/day)	21 days	Inflammation cytokines; Microvascular function	**Effective.** Reduced IL-17A and pro-inflammatory mediators (2022); Enhanced endothelium-dependent vasodilation via COX-2 pathways (2023).
(Nouhravesh et al. 2025) (USA)	RCT, 2x2 Factorial	n=140; CVD risk	Fortified eggs vs. No eggs; Time-restricted eating (TRE)	4 months	Lipids, Cardiometabolic markers	**Null.** No clinical difference in lipid profiles between fortified egg consumption and egg restriction.

Note: This table is organized by unique study, not by publication count. Therefore, it includes 37 study entries. Ata et al. (2010/2011) and Šušnjara et al. (2022/2023) represent paired publications from the same underlying studies and were merged into single study entries. Accordingly, the reference list contains 39 publications, whereas Table S1 contains 37 studies.

Abbreviations: ALA: alpha-linolenic acid; ApoB: apolipoprotein B; BP: blood pressure; COX-2: cyclooxygenase-2; CRP: C-reactive protein; CVD: cardiovascular disease; DBP: diastolic blood pressure; DHA: docosahexaenoic acid; EPA: eicosapentaenoic acid; HDL-C: high-density lipoprotein cholesterol; HOMA-IR: Homeostatic Model Assessment of Insulin Resistance; hsCRP: high-sensitivity C-reactive protein; IgG: immunoglobulin G; IL: interleukin; INF-γ: interferon-gamma; LDL-C: low-density lipoprotein cholesterol; MetS: metabolic syndrome; MPOD: macular pigment optical density; n-3 PUFA: omega-3 polyunsaturated fatty acids; RBC: red blood cell; RCT: randomized controlled trial; ROS: reactive oxygen species; SBP: systolic blood pressure; SOD: superoxide dismutase; TBARS: thiobarbituric acid reactive substances; TC: total cholesterol; TG: triglycerides; TMAO: trimethylamine N-oxide; TRE: time-restricted eating; UIC: urinary iodine concentration.

Table S1 References

1. Adabi, S. H. G., M. A. Kamali, J. Davoudi, R. G. Cooper, and A. Hajbabaei. 2010. "Quantification of Lutein in Egg Following Feeding Hens with a Lutein Supplement and Quantification of Lutein in Human Plasma after Consumption of Lutein Enriched Eggs." *Archiv Für Geflügelkunde* 74 (3): 158–63. https://doi.org/10.1016/s0003-9098(25)00813-6.

2. Ata, Shymaa, J. Barona, R. Kopec, J. Jones, M. Calle, S. Schwartz, and M. L. Fernandez. 2010. "Consumption of Either One Egg or Lutein-Enriched Egg per Day Increases HDL Cholesterol, Reduces Apolipoprotein B While Increasing Plasma Carotenoids and Macular Pigment Density in Adult Subjects." *FASEB Journal* 24 (S1): 92.4. https://doi.org/10.1096/fasebj.24.1_supplement.92.4.

3. Ata, Shymaa, J. P. Barona, and Maria Luz Fernández. 2011. "Consumption of One Lutein-enriched Egg per Day Decreases C Reactive Protein, Plasma Insulin Levels and Improves Insulin Sensitivity in Men and Women." *FASEB Journal* 25 (S1). https://doi.org/10.1096/fasebj.25.1_supplement.975.2.

4. Azgar, S., Y. Xu, S. Kalia, T. Sun, K. J. Ou, X. G. Lei, and K. O'Brien. 2024. "Bioavailability of DHA from Bioenhanced Eggs Is Greater than That Observed from Bioenhanced Poultry in Healthy Adults." *Current Developments in Nutrition* 8 (Supplement 2): 102857. https://doi.org/10.1016/j.cdnut.2024.102857.

5. Bovet, P., D. Faeh, G. Madeleine, B. Viswanathan, and F. Paccaud. 2007. "Decrease in Blood Triglycerides Associated with the Consumption of Eggs of Hens Fed with Food Supplemented with Fish Oil." *Nutrition, Metabolism and Cardiovascular Diseases* 17 (4): 280–87. https://doi.org/10.1016/j.numecd.2005.12.010.

6. Burns-Whitmore, Bonny, E. H. Haddad, J. Sabaté, K. Jaceldo-Siegl, J. Tanzman, and S. Rajaram. 2010. "Effect of N-3 Fatty Acid Enriched Eggs and Organic Eggs on Serum Lutein in Free-Living Lacto-Ovo Vegetarians." *European Journal of Clinical Nutrition* 64 (11): 1332–37. https://doi.org/10.1038/ejcn.2010.140.

7. Burns-Whitmore, Bonny, E. Haddad, J. Sabaté, and S. Rajaram. 2014. "Effects of Supplementing N-3 Fatty Acid Enriched Eggs and Walnuts on Cardiovascular Disease Risk Markers in Healthy Free-Living Lacto-Ovo-Vegetarians: A Randomized, Crossover, Free-Living Intervention Study." *Nutrition Journal* 13 (1): 29. https://doi.org/10.1186/1475-2891-13-29.

8. Charoensiriwatana, W., P. Srijantr, P. Teeyapant, and J. Wongvilairattana. 2010.

"Consuming Iodine Enriched Eggs to Solve the Iodine Deficiency Endemic for Remote Areas in Thailand." *Nutrition Journal* 9 (1): 68. https://doi.org/10.1186/1475-2891-9-68.

9. Drenjancevic, I., A. Stupin, P. Susnjara, N. Kolobaric, L. Kolar, A. Marija Masle, Z. B. Curic, et al. 2022. "Does Functional Food Provide Benefits for Cardiovascular Health? Ongoing Studies in Scientific Centre of Excellence." *Journal of Hypertension* 40 (Suppl 1): e206. https://doi.org/10.1097/01.hjh.0000837544.05525.4c.

10. Fakhrzadeh, H., R. Poorebrahim, P. Shooshtarizadeh, M. Raza, and S. Hosseini. 2005. "The Effects of Consumption of Ω3 Fatty Acid-Enriched Eggs on Insulin and CRP." *Nutrition Metabolism and Cardiovascular Diseases* 15 (4): 329–30. https://doi.org/10.1016/j.numecd.2004.12.004.

11. Galvis, Y., K. Pineda, J. Zapata, J. Aristizabal, A. Estrada, M. L. Fernandez, and J. Barona-Acevedo. 2023. "Consumption of Eggs Alone or Enriched with Annatto (Bixa Orellana L.) Does Not Increase Cardiovascular Risk in Healthy Adults-a Randomized Clinical Trial, the Eggant Study." *Nutrients* 15 (2). https://doi.org/10.3390/nu15020369.

12. Gillingham, L. G., L. Caston, S. Leeson, K. Hourtovenko, and B. J. Holub. 2005. "The Effects of Consuming Docosahexaenoic Acid (DHA)-Enriched Eggs on Serum Lipids and Fatty Acid Compositions in Statin-Treated Hypercholesterolemic Male Patients." *Food Research International* 38 (10): 1117–23. https://doi.org/10.1016/j.foodres.2005.03.006.

13. Grønborg, I. M., I. Tetens, T. Christensen, E. W. Andersen, J. Jakobsen, M. Kiely, K. D. Cashman, and R. Andersen. 2020. "Vitamin D-Fortified Foods Improve Wintertime Vitamin D Status in Women of Danish and Pakistani Origin Living in Denmark: A Randomized Controlled Trial." *European Journal of Nutrition* 59 (2): 741–53. https://doi.org/10.1007/s00394-019-01941-6.

14. Hegde, M. V., A. A. Zanwar, S. A. Khan, and U. V. Wagh. 2011. "Omega-3 Fatty Acid Enriched Eggs Are Heart Friendly." *Atherosclerosis Supplements* 12 (1): 148. https://doi.org/10.1016/s1567-5688(11)70706-1.

15. Hu, Weili, Shuqi Li, Xuecong Li, Amelia Shan Mei Chng, Chunjun Quek, Zhihui Chong, Chin Meng Khoo, and Jung Eun Kim. 2024. "Impact of Consumption of Antioxidant Fortified Eggs on Eye Health: A Randomized Controlled Trial." *Current Developments in Nutrition* 8 (Supplement 2): 102250.

https://doi.org/10.1016/j.cdnut.2024.102250.

16. Imran, M., F. M. Anjum, M. S. Butt, and Z. A. Chatha. 2014. "Incorporation of Nutritionally Important Fatty Acids into Eggs and Evaluation of ‘Bio-Omega-3’ Eggs in Humans with Moderate Hypercholesterolemia." *Pakistan Journal of Nutrition* 12 (10): 907–11. https://doi.org/10.3923/pjn.2013.907.911.

17. Jaček, Martin, Dana Hrnčířová, Jolana Rambousková, Pavel Dlouhý, and Petr Tůma. 2020. "Effect of Food with Low Enrichment of N-3 Fatty Acids in a Two-Month Diet on the Fatty Acid Content in the Plasma and Erythrocytes and on Cardiovascular Risk Markers in Healthy Young Men." *Nutrients* 12 (8): 2207. https://doi.org/10.3390/nu12082207.

18. Kelly, D., J. M. Nolan, A. N. Howard, J. Stack, K. O. Akuffo, R. Moran, D. I. Thurnham, J. Dennison, K. A. Meagher, and S. Beatty. 2017. "Serum and Macular Response to Carotenoid-Enriched Egg Supplementation in Human Subjects: The Egg Xanthophyll Intervention Clinical Trial (EXIT)." *British Journal of Nutrition* 117 (1): 108–23. https://doi.org/10.1017/S0007114516003895.

19. Kelly, E. R., J. Plat, G. R. M. M. Haenen, A. Kijlstra, and T. T. J. M. Berendschot. 2014. "The Effect of Modified Eggs and an Egg-Yolk Based Beverage on Serum Lutein and Zeaxanthin Concentrations and Macular Pigment Optical Density: Results from a Randomized Trial." *PLOS One* 9 (3): e92659. https://doi.org/10.1371/journal.pone.0092659.

20. Kolar, L., M. Stupin, A. Stupin, P. Susnjara, Z. Mihaljevic, A. Matic, I. Jukic, N. Kolobaric, and I. Drenjancevic. 2021. "Does the Endothelium of Competitive Athletes Benefit from Consumption of N-3 Polyunsaturated Fatty Acid-Enriched Hen Eggs?" *Preventive Nutrition and Food Science* 26 (4): 388–99. https://doi.org/10.3746/pnf.2021.26.4.388.

21. Kolar, L., P. Susnjara, M. Stupin, A. Stupin, I. Jukic, Z. Mihaljevic, N. Kolobaric, et al. 2023. "Enhanced Microvascular Adaptation to Acute Physical Stress and Reduced Oxidative Stress in Male Athletes Who Consumed Chicken Eggs Enriched with N-3 Polyunsaturated Fatty Acids and Antioxidants-Randomized Clinical Trial." *Life-Basel* 13 (11). https://doi.org/10.3390/life13112140.

22. Kolobarić, N., A. Matić, P. Šušnjara, M. Mihalj, and I. Drenjancevic. 2021. "N-3-Polyunsaturated Fatty Acids-Enriched Eggs Consumption Increases Serum Concentrations of E-Series Resolvins in Young Healthy Participants." *Annals of Nutrition & Metabolism* 77 (Suppl. 1): 11. https://doi.org/10.1159/000518127.

23. Lu, Y., Y. J. Hao, X. Zhou, F. Huang, C. Li, J. Wang, Z. Miao, et al. 2024. “Effects of Long-Term Intake of Carotenoid-Enriched Eggs on Healthy People: A Randomized Controlled Study.” *Food & Function* 15 (13): 7032–45. https://doi.org/10.1039/d4fo00910j.

24. Made, Sanne M. van der, Elton R. Kelly, Tos T. J. M. Berendschot, Aize Kijlstra, Dieter Lütjohann, and Jogchum Plat. 2014. “Consuming a Buttermilk Drink Containing Lutein-Enriched Egg Yolk Daily for 1 Year Increased Plasma Lutein but Did Not Affect Serum Lipid or Lipoprotein Concentrations in Adults with Early Signs of Age-Related Macular Degeneration.” *The Journal of Nutrition* 144 (9): 1370–77. https://doi.org/10.3945/jn.114.195503.

25. Mihalj, M., A. Stupin, N. Kolobarić, I. T. Bujak, A. Matić, Z. Kralik, I. Jukić, M. Stupin, and I. Drenjančević. 2020. “Leukocyte Activation and Antioxidative Defense Are Interrelated and Moderately Modified by N-3 Polyunsaturated Fatty Acid-Enriched Eggs Consumption—Double-Blind Controlled Randomized Clinical Study.” *Nutrients* 12 (10): 1–21. https://doi.org/10.3390/nu12103122.

26. Njembe, M. T. N., B. Pachikian, I. Lobysheva, N. Van Overstraeten, L. Dejonghe, E. Verstraelen, M. Buchet, et al. 2021. “A Three-Month Consumption of Eggs Enriched with ω-3, ω-5 and ω-7 Polyunsaturated Fatty Acids Significantly Decreases the Waist Circumference of Subjects at Risk of Developing Metabolic Syndrome: A Double-Blind Randomized Controlled Trial.” *Nutrients* 13 (2): 1–19. https://doi.org/10.3390/nu13020663.

27. Nouhravesh, Nina, Josephine Harrington, Laura H. Aberle, Cynthia L. Green, Kathleen Voss, Dave Holdsworth, Kurt Misialek, et al. 2025. “Effects of Fortified Eggs and Time-Restricted Eating on Cardiometabolic Health: The Prosperity Trial.” *American Heart Journal* 279 (2025): 27–39. https://doi.org/10.1016/j.ahj.2024.10.005.

28. Ohman, M., T. Akerfeldt, I. Nilsson, C. Rosen, L. O. Hansson, M. Carlsson, and A. Larsson. 2008. “Biochemical Effects of Consumption of Eggs Containing Omega-3 Polyunsaturated Fatty Acids.” *Upsala Journal of Medical Sciences* 113 (3): 315–23. https://doi.org/10.3109/2000-1967-235.

29. Radanović, Ana, Gordana Kralik, Ines Drenjančević, Olivera Galović, Manuela Košević, and Zlata Kralik. 2023. “N-3 PUFA Enriched Eggs as a Source of Valuable Bioactive Substances.” *Foods* 12 (23): 4202. https://doi.org/10.3390/foods12234202.

30. Rasic, L., A. Cosic, Z. Kralik, G. Kralik, A. Cavka, and I. Drenjancevic. 2015. "Effect of Omega-3 Enriched Food on Oxidative Stress Levels in Young Healthy Men." *Journal of Hypertension* 33 (e-Supplement 1): e147–48. https://doi.org/10.1097/01.hjh.0000467771.42714.4e.

31. Schnebelen-Berthier, Coralie, Niyazi Acar, Emilie Simon, Clémentine Thabuis, Anne Bourdillon, Adeline Mathiaud, Luc Dauchet, et al. 2021. "The ALGOVUE Clinical Trial: Effects of the Daily Consumption of Eggs Enriched with Lutein and Docosahexaenoic Acid on Plasma Composition and Macular Pigment Optical Density." *Nutrients* 13 (10): 3347. https://doi.org/10.3390/nu13103347.

32. Shakoor, H., M. I. Khan, A. Sahar, M. K. I. Khan, F. Faiz, and H. B. Ahmad. 2020. "Development of Omega-3 Rich Eggs through Dietary Flaxseed and Bio-Evaluation in Metabolic Syndrome." *Food Science & Nutrition* 8 (6): 2619–26. https://doi.org/10.1002/fsn3.1522.

33. Stanton, Alice V., Kirstyn James, Margaret M. Brennan, Fiona O'Donovan, Fahad Buskandar, Kathleen Shortall, Thora El-Sayed, et al. 2020. "Omega-3 Index and Blood Pressure Responses to Eating Foods Naturally Enriched with Omega-3 Polyunsaturated Fatty Acids: A Randomized Controlled Trial." *Scientific Reports* 10 (1): 15444. https://doi.org/10.1038/s41598-020-71801-5.

34. Stupin, A., A. Cvetko, G. Kralik, M. Mihalj, P. Susnjara, N. Kolobaric, Z. B. Curic, et al. 2021. "The Effect of N-3 Polyunsaturated Fatty Acids-Enriched Hen Eggs Consumption on IgG and Total Plasma Protein N-Glycosylation in Healthy Individuals and Cardiovascular Patients." *Glycobiology* 31 (9): 1163–75. https://doi.org/10.1093/glycob/cwab051.

35. Stupin, A., M. Mihalj, N. Kolobaric, P. Susnjara, L. Kolar, Z. Mihaljevic, A. Matic, et al. 2020. "Anti-Inflammatory Potential of n-3 Polyunsaturated Fatty Acids Enriched Hen Eggs Consumption in Improving Microvascular Endothelial Function of Healthy Individuals-Clinical Trial." *International Journal of Molecular Sciences* 21 (11). https://doi.org/10.3390/ijms21114149.

36. Stupin, A., L. Rasic, A. Matic, M. Stupin, Z. Kralik, G. Kralik, M. Grcevic, and I. Drenjancevic. 2018. "Omega-3 Polyunsaturated Fatty Acids-Enriched Hen Eggs Consumption Enhances Microvascular Reactivity in Young Healthy Individuals." *Applied Physiology, Nutrition, and Metabolism* 43 (10): 988–95. https://doi.org/10.1139/apnm-2017-0735.

37. Šušnjara, P., N. Kolobarić, A. Matić, Z. Mihaljević, A. Stupin, S. Marczi, and I.

Drenjančević. 2022. “Consumption of Hen Eggs Enriched with N-3 Polyunsaturated Fatty Acids, Selenium, Vitamin E and Lutein Incites Anti-Inflammatory Conditions in Young, Healthy Participants - a Randomized Study.” *Frontiers in Bioscience-Landmark* 27 (12): 332. https://doi.org/10.31083/j.fbl2712332.

38. Šušnjara, P., Z. Mihaljević, A. Stupin, N. Kolobarić, A. Matić, I. Jukić, Z. Kralik, et al. 2023. “Consumption of Nutritionally Enriched Hen Eggs Enhances Endothelium-Dependent Vasodilation via Cyclooxygenase Metabolites in Healthy Young People-a Randomized Study.” *Nutrients* 15 (7). https://doi.org/10.3390/nu15071599.

39. West, Allyson A., Yun Shih, Wei Wang, Keiji Oda, Karen Jaceldo-Siegl, Joan Sabaté, Ella Haddad, Sujatha Rajaram, Marie A. Caudill, and Bonny Burns-Whitmore. 2014. “Egg N-3 Fatty Acid Composition Modulates Biomarkers of Choline Metabolism in Free-Living Lacto-Ovo-Vegetarian Women of Reproductive Age.” *Journal of the Academy of Nutrition and Dietetics* 114 (10): 1594–600. https://doi.org/10.1016/j.jand.2014.02.012.

Section S3: List of Excluded Studies

A total of **75** articles were assessed for eligibility at the full-text level but were excluded. The citations are grouped below by the primary reason for exclusion.

Reason 1: Market research or consumer surveys (n = 38)

1. Maksan, M. T., Z. Mesic, B. S. Bobic, et al. 2025. "Understanding consumers' willingness to use omega-3 enriched eggs." Economia Agro-alimentare. doi:10.3280/ecag2025oa18685.
2. Areal, F. J., and D. Asioli. 2024. "Heterogeneous preferences and consumer willingness to pay for vitamin D fortification of eggs." Agribusiness 40 (3): 661–679.
3. Kukec, A., V. Vicic, and R. P. Mikus. 2023. "Cost comparison of vitamin D from egg biofortification: the case of Slovenia study." European Journal of Public Health 33.
4. Crncan, A., S. J. Milkovic, M. Jaksic, et al. 2023. "Competitive analysis of omega-3 polyunsaturated fatty acid-enriched eggs in the republic of Croatia." World's Poultry Science Journal. doi:10.1080/00439339.2023.2250338.
5. Chen, R., C. Jiang, X. Li, et al. 2023. "Research on Chinese consumers' shell egg consumption preferences and the egg quality of functional eggs." Poultry Science 102 (10): 103007.
6. Tian, Y. X., H. Zhu, L. Zhang, et al. 2022. "Consumer preference for nutritionally fortified eggs and impact of health benefit information." Foods 11 (8): 1145.
7. Panzone, L., G. Garrod, F. Adinolfi, et al. 2022. "Molecular marketing, personalised information and willingness-to-pay for functional foods: vitamin D enriched eggs." Journal of Agricultural Economics 73 (3): 666–689.
8. Palmieri, N., W. Stefanoni, F. Latterini, et al. 2022. "Factors influencing Italian consumers' willingness to pay for eggs enriched with omega-3-fatty acids." Foods 11 (4): 545.
9. Migliore, G., G. Rizzo, A. Bonanno, et al. 2022. "Functional food characteristics in organic food products-the perspectives of Italian consumers on organic eggs enriched with omega-3 polyunsaturated fatty acids." Organic Agriculture 12 (2): 149–161.
10. Zhu, Hong, Kehong Liang, Yifen Hong, et al. 2021. "Interpretation of the agricultural industry standard @omega-3 polyunsaturated fatty acid fortified eggs'." China Standardization 19: 196–199, 204. (in Chinese)

11. Xiao, Haixia. 2021. Yuntianshi agricultural technology health egg project. (in Chinese)
12. Peng, Zhiwei, Xiaoting Liu, and Qingping Wang. 2021. “Application research of functional egg yolk powder replacing antibiotics in piglet feed.” Hunan Feed 3: 38–40. (in Chinese)
13. Shi, Dayou. 2019. Application demonstration of antibiotic-free and health-functional egg production technology. (in Chinese)
14. Chai, Yu, and Na Liu. 2019. “Cooking eggs in various ways.” Chinese Medicine Health Preservation 5 (9): 12–15. (in Chinese)
15. Baba, Y., Z. Kallas, and C. Realini. 2017. “Application of the analytical hierarchy process to evaluate consumer acceptance and preferences for omega-3 enriched eggs.” British Food Journal 119 (7): 1459–1472.
16. Xie, Qian. 2015. “Establishment of HPLC-ICP/MS method for detection of selenium in selenium-fortified eggs.” Master’s thesis, Huazhong Agricultural University. (in Chinese)
17. Liu, Jingbo. 2014. Industrial production demonstration of functional poultry egg products. (in Chinese)
18. Zhu, Ling. 2012. “Dazzling ‘functional eggs’.” Health and Life 4: 14. (in Chinese)
19. Yang, Zai, Wei Xiao, Hengyu Yin, et al. 2012. “Market potential analysis of special chickens and functional eggs.” Rural Breeding Technology 10: 44. (in Chinese)
20. Yang, Zai, Wei Xiao, Hengyu Lyu, et al. 2012. “How big can the market for special chickens and functional eggs be?” Guide to Chinese Poultry 29 (4): 19. (in Chinese)
21. Ren, Xiao. 2012. “‘Functional eggs’, don’t be ridiculous.” Shanghai Department Store 3: 7. (in Chinese)
22. Sheehy, T., and S. Sharma. 2011. “Use of FAO food balance sheets to estimate the potential ability of novel folate-enriched eggs to increase the folate supply in European Union countries.” Public Health Nutrition 14 (3): 551–556.
23. Xiao, Wei. 2011. “Functional eggs and organic vegetables are mostly unreliable.” Self Health Preservation 12: 14–15. (in Chinese)
24. Xiao, Wei. 2011. “Hyping health concepts: functional eggs and organic vegetables are mostly unreliable.” Self Health Preservation 12: 14–15. (in Chinese)
25. Feng, Guangde. 2011. Research and demonstration of layer industrialization technology. (in Chinese)
26. Hayat, Z., T. N. Pasha, F. M. Khattak, et al. 2010. “Consumer’s perception and

willingness to buy nutrient enriched designer eggs in Pakistan.” Archiv für Geflügelkunde 74 (3): 145–150.
27. Liu, Rong. 2010. Eggs rich in conjugated linoleic acid. (in Chinese)
28. Lin, Xiao. 2010. “Nutritional functional eggs—worm eggs.” Technology and Market 17 (2): 85. (in Chinese)
29. Research on Sanjia Kangle eggs. 2009. (in Chinese)
30. Zhuan, Xiliang. 2009. “Academician Wu Changxin jokes about ‘native eggs’ and ‘functional eggs’.” Northern Animal Husbandry 7: 10. (in Chinese)
31. Zhi, Yongping, and Fubin Han. 2008. “Breakthrough in breeding and production technology of pink-shell laying hens in Shaanxi Province.” Beijing Agriculture 22: 38. (in Chinese)
32. Zhi, Yongping. 2008. “Breakthrough in pink-shell layer breeding and health functional egg research in our province.” Shaanxi Science and Technology News, March 21, 1. (in Chinese)
33. Xia, Ji. 2006. “Introduction to nutritional health eggs.” Rural Practical Science and Technology Information 4: 28. (in Chinese)
34. “Green-shell laying hens.” 2005. Farm Wealth 18: 38. (in Chinese)
35. Development of Zhikang eggs with high content of DHA and EPA. 2005. (in Chinese)
36. Yue, Guangzhi. 2005. “Health eggs? Have personality!” Modern Health Care 11: 40–41. (in Chinese)
37. Wen, Huan. 2005. “Cholesterol-lowering eggs come out.” Cereals and Oils Food Science and Technology 13 (2): 48. (in Chinese)
38. Sun, Daode. 2005. “Cultivating ‘health eggs’ with complete color and fragrance.” Rural Practical Technology and Information 11: 55–56. (in Chinese)

Reason 2: Reviews or secondary research (n = 25)

39. Yu, Yanan, Kehan Ma, Jia Yang, et al. 2025. "Research progress on bio-fortified eggs." *Scientia Sinica Vitae* 55 (3): 489–507. (in Chinese)
40. Sultan, N., E. Cheng, C. McMahon, et al. 2024. "The impact of egg consumption on cognitive function: a systematic literature review." *Proceedings of the Nutrition Society* 83 (OCE1): E181.
41. Cashman, K. D., and C. M. O’Neill. 2024. "Strategic food vehicles for vitamin D fortification and effects on vitamin D status: a systematic review and meta-analysis of randomised controlled trials." *Journal of Steroid Biochemistry and Molecular Biology* 238: 106448.
42. Wang, Hui, and Fenglong Cai. 2024. "Storage stability and antioxidant strategies

of omega-3 polyunsaturated fatty acid fortified eggs." *Livestock Environment* 21: 13–15. (in Chinese)
43. Qian, Yong. 2024. "Trend analysis and development difficulties of functional egg products." *Guide to Chinese Poultry* 41 (5): 1. (in Chinese)
44. Dansou, D. M., H. Zhang, Y. Yu, et al. 2023. "Carotenoid enrichment in eggs: from biochemistry perspective." *Animal Nutrition* 14: 315–333.
45. Zhang, Qiufang, Ziyi Wang, and Xiaofang Shen. 2023. "Research progress on DHA fortified eggs." *China Oils and Fats* 48: 98–103. (in Chinese)
46. Jiang, Caiyun, Xuefeng Shi, Ruochen Chen, et al. 2023. "Research progress on omega-3 polyunsaturated fatty acid fortified eggs." *China Poultry* 45: 82–89. (in Chinese)
47. Zhu, Liping, Jianchuan Zhou, and Honglin Yan. 2022. "Research progress on lutein fortified eggs." *Animal Husbandry Industry* 3: 62–71. (in Chinese)
48. Zhu, Hong, Kehong Liang, Guangran Li, et al. 2022. "Research progress on omega-3 polyunsaturated fatty acid fortified eggs: production, quality and health effects." *Food and Nutrition in China* 28 (9): 38–43. (in Chinese)
49. Zheng, Yan, Qingcui Xu, Lixia Fan, et al. 2022. "Research progress on evaluation of egg nutritional quality." *Food and Nutrition in China* 28 (1): 45–50. (in Chinese)
50. Kralik, Z., G. Kralik, M. Grcevic, et al. 2021. "Designer eggs with an increased content of omega-3 fatty acids and pigments - production and health benefits of their consumption." *Poljoprivreda* 27 (2): 67–74.
51. Wang, Hao, Qingyu Zhao, Junmin Zhang, et al. 2020. "Research progress on storage stability of omega-3 polyunsaturated fatty acid fortified eggs." *Chinese Journal of Animal Nutrition* 32: 2959–2965. (in Chinese)
52. Rodríguez-Rodríguez, E., A. Aparicio Vizuete, P. Sánchez-Rodríguez, et al. 2019. "[Vitamin D deficiency in Spanish population. Importance of egg on nutritional improvement]." *Nutricion Hospitalaria* 36 (Spec No3): 3–7.
53. Papierska, K., and E. Ignatowicz. 2019. "Functional food in prevention of cardiovascular diseases and obesity." *Acta Poloniae Pharmaceutica - Drug Research* 76 (6): 945–958.
54. Nimalaratne, C., and J. Wu. 2015. "Hen egg as an antioxidant food commodity: a review." *Nutrients* 7 (10): 8274–8293.
55. Miranda, J. M., X. Anton, C. Redondo-Valbuena, et al. 2015. "Egg and egg-derived foods: effects on human health and use as functional foods." *Nutrients* 7 (1): 706–729.
56. Zhou, Ying, and Zhili Qi. 2015. "Research progress on regulation of egg quality

by polyunsaturated fatty acids." *China Poultry* 37: 48–51. (in Chinese)
57. Xu, Guiyun. 2012. "New understanding of egg quality and nutritional value." *China Poultry* 34 (13): 36–38. (in Chinese)
58. Yalçin, H., and M. K. Ünal. 2010. "The enrichment of hen eggs with ω-3 fatty acids." *Journal of Medicinal Food* 13 (3): 610–614.
59. Shapira, N. 2009. "Modified egg as a nutritional supplement during peak brain development: a new target for fortification." *Nutrition and Health* 20 (2): 107–118.
60. Fisinin, V. I., T. T. Papazyan, and P. F. Surai. 2009. "Producing selenium-enriched eggs and meat to improve the selenium status of the general population." *Critical Reviews in Biotechnology* 29 (1): 18–28.
61. Fisinin, V. I., T. T. Papazyan, and P. F. Surai. 2008. "Producing specialist poultry products to meet human nutrition requirements: selenium enriched eggs." *World's Poultry Science Journal* 64 (1): 85–97.
62. Cui, Jia, Shaoyu Li, and Bin Xu. 2007. "Research progress on nutrition of polyunsaturated fatty acid fortified eggs." *Journal of Henan Agricultural Sciences* 3: 114–116. (in Chinese)
63. Novello, D., P. Franceschini, D. A. Quintiliano, et al. 2006. "[Egg: concepts, analyses and controversies in the human health]." *Archivos Latinoamericanos de Nutrición* 56 (4): 315–320.

Reason 3: Duplicate data or redundant abstracts (Final full-text already included) (n = 10)

64. Burns-Whitmore, B., S. Rajaram, E. Haddad, et al. 2005. "Effect of n-3 fatty acid enriched eggs vs. walnuts on blood lipids in free-living lacto-ovo vegetarians." The FASEB Journal 19 (5): A1011–A1012. (Abstract of Burns-Whitmore 2010).
65. Ata, Shymaa, J. P. Barona, and M. L. Fernandez. 2011. "Consumption of one lutein-enriched egg per day decreases CRP and plasma insulin." The FASEB Journal 25. (Preliminary data of full trial included).
66. Kelly, D., J. M. Nolan, A. N. Howard, et al. 2016. "The Egg Xanthophyll Intervention Clinical Trial (EXIT)." Proceedings of the Nutrition Society. (Abstract of Kelly 2017).
67. Jaček, M., D. Hrnčířová, and P. Tůma. 2019. "Dietary intake of n–3 fatty acids from enriched eggs." Clinical Nutrition ESPEN. (Abstract of Jaček 2020).
68. Nouhravesh, N., J. Harrington, et al. 2024. "Prospective evaluation of fortified

eggs: Primary results from the Prosperity Trial." Journal of the American College of Cardiology. (Abstract of Nouhravesh 2025).

69. van der Made, S. M., et al. 2013. "Increased plasma lutein after fortified egg consumption." The Journal of Nutrition. (Preliminary results of van der Made 2014).
70. Adabi, S. H. G., et al. 2009. "Lutein enriched eggs and human plasma response." British Poultry Science. (Conference paper of Adabi 2010).
71. Radanović, A., et al. 2022. "N-3 PUFA enriched eggs as functional food." Foods. (Preliminary study of Radanović 2023).
72. Charoensiriwatana, W., et al. 2009. "Iodine enriched eggs for IDD control." Nutrition Journal. (Preliminary abstract of Charoensiriwatana 2010).
73. Lu, Y., et al. 2023. "Long-term intake of carotenoid-enriched eggs." Food & Function. (Early access version of Lu 2024).

Reason 4: Database registration error (n = 1)

74. Ferrier, L. K., L. J. Caston, S. Leeson, J. Squires, B. J. Weaver, and B. J. Holub. 1995. "α-Linolenic acid- and docosahexaenoic acid-enriched eggs from hens fed flaxseed: influence on blood lipids and platelet phospholipid fatty acids in humans." The American Journal of Clinical Nutrition 62 (1): 81–86.

Reason 5: Unable to retrieve an article (n = 1)

75. Burns-Whitmore, B., S. Rajaram, E. Haddad, et al. 2005. "Effect of n-3 fatty acid enriched eggs vs. walnuts on blood lipids in free-living lacto-ovo vegetarians." The FASEB Journal 19 (5): A1011–A1012.

Section S4: PRISMA-ScR Checklist

Preferred Reporting Items for Systematic reviews and Meta-Analyses extension for Scoping Reviews (PRISMA-ScR) Checklist

SECTION	ITEM	PRISMA-ScR CHECKLIST ITEM	REPORTED ON PAGE #
**TITLE**			
Title	1	Identify the report as a scoping review.	Title page
**ABSTRACT**			
Structured summary	2	Provide a structured summary that includes (as applicable): background, objectives, eligibility criteria, sources of evidence, charting methods, results, and conclusions that relate to the review questions and objectives.	Page 1
**INTRODUCTION**			
Rationale	3	Describe the rationale for the review in the context of what is already known. Explain why the review questions/objectives lend themselves to a scoping review approach.	Page 2
Objectives	4	Provide an explicit statement of the questions and objectives being addressed with reference to their key elements (e.g., population or participants, concepts, and context) or other relevant key elements used to conceptualize the review questions and/or objectives.	Page 2
**METHODS**			
Protocol and registration	5	Indicate whether a review protocol exists; state if and where it can be accessed (e.g., a Web address); and if available, provide registration information, including the registration number.	Page 3
Eligibility criteria	6	Specify characteristics of the sources of evidence used as eligibility criteria (e.g., years considered, language, and publication status), and provide a rationale.	Page 3
Information sources*	7	Describe all information sources in the search (e.g., databases with dates of coverage and contact with authors to	Page 4

SECTION	ITEM	PRISMA-ScR CHECKLIST ITEM	REPORTED ON PAGE #
		identify additional sources), as well as the date the most recent search was executed.	
Search	8	Present the full electronic search strategy for at least 1 database, including any limits used, such that it could be repeated.	Page 3 and Appendix A
Selection of sources of evidence†	9	State the process for selecting sources of evidence (i.e., screening and eligibility) included in the scoping review.	Page 3
Data charting process‡	10	Describe the methods of charting data from the included sources of evidence (e.g., calibrated forms or forms that have been tested by the team before their use, and whether data charting was done independently or in duplicate) and any processes for obtaining and confirming data from investigators.	Page 3-4
Data items	11	List and define all variables for which data were sought and any assumptions and simplifications made.	Page 4
Critical appraisal of individual sources of evidence§	12	If done, provide a rationale for conducting a critical appraisal of included sources of evidence; describe the methods used and how this information was used in any data synthesis (if appropriate).	Not Applicable
Synthesis of results	13	Describe the methods of handling and summarizing the data that were charted.	Page 4
**RESULTS**			
Selection of sources of evidence	14	Give numbers of sources of evidence screened, assessed for eligibility, and included in the review, with reasons for exclusions at each stage, ideally using a flow diagram.	Page 4, Figure 1 and Appendix C
Characteristics of sources of evidence	15	For each source of evidence, present characteristics for which data were charted and provide the citations.	Table 1 and Appendix B
Critical appraisal within sources of evidence	16	If done, present data on critical appraisal of included sources of evidence (see item 12).	Not Applicable
Results of individual	17	For each included source of evidence, present the relevant data that were charted	Appendix B

SECTION	ITEM	PRISMA-ScR CHECKLIST ITEM	REPORTED ON PAGE #
sources of evidence		that relate to the review questions and objectives.	
Synthesis of results	18	Summarize and/or present the charting results as they relate to the review questions and objectives.	Table 2
**DISCUSSION**			
Summary of evidence	19	Summarize the main results (including an overview of concepts, themes, and types of evidence available), link to the review questions and objectives, and consider the relevance to key groups.	Page 8
Limitations	20	Discuss the limitations of the scoping review process.	Page 9-10
Conclusions	21	Provide a general interpretation of the results with respect to the review questions and objectives, as well as potential implications and/or next steps.	Page 10
**FUNDING**			
Funding	22	Describe sources of funding for the included sources of evidence, as well as sources of funding for the scoping review. Describe the role of the funders of the scoping review.	Page 10

JBI = Joanna Briggs Institute; PRISMA-ScR = Preferred Reporting Items for Systematic reviews and Meta-Analyses extension for Scoping Reviews.

* Where *sources of evidence* (see second footnote) are compiled from, such as bibliographic databases, social media platforms, and Web sites.

† A more inclusive/heterogeneous term used to account for the different types of evidence or data sources (e.g., quantitative and/or qualitative research, expert opinion, and policy documents) that may be eligible in a scoping review as opposed to only studies. This is not to be confused with *information sources* (see first footnote).

‡ The frameworks by Arksey and O'Malley (6) and Levac and colleagues (7) and the JBI guidance (4, 5) refer to the process of data extraction in a scoping review as data charting.

§ The process of systematically examining research evidence to assess its validity, results, and relevance before using it to inform a decision. This term is used for items 12 and 19 instead of "risk of bias" (which is more applicable to systematic reviews of interventions) to include and acknowledge the various sources of evidence that may be used in a scoping review (e.g., quantitative and/or qualitative research, expert opinion, and policy document).

From: Tricco AC, Lillie E, Zarin W, O'Brien KK, Colquhoun H, Levac D, et al. PRISMA Extension for Scoping Reviews (PRISMAScR): Checklist and Explanation. Ann Intern Med. 2018;169:467–473. doi: 10.7326/M18-0850.
